# From Ethnopharmacology to Active Compound: Effects of Traditional Plant Extracts on Varicose Vein-Related Enzymes and Isolation of Active Flavonoids from *Helichrysum plicatum* DC. subsp. *plicatum*

**DOI:** 10.3390/ph18060926

**Published:** 2025-06-19

**Authors:** Tugsen Buyukyildirim, F. Sezer Senol Deniz, Merve Yuzbasioglu Baran, Ece Salihoglu, Mustafa Abdullah Yilmaz, Osman Tugay

**Affiliations:** 1 Department of Pharmacognosy, Faculty of Pharmacy, Selcuk University, 42071 Konya, Türkiye; tugsen.dogru@selcuk.edu.tr; 2 Department of Pharmacognosy, Faculty of Pharmacy, Gazi University, 06330 Ankara, Türkiye; 3 Department of Pharmacognosy, Gülhane Faculty of Pharmacy, University of Health Sciences, 06108 Ankara, Türkiye; merve.yuzbasioglu@sbu.edu.tr; 4 Department of Biochemistry, Faculty of Pharmacy, Gazi University, 06330 Ankara, Türkiye; ecemiser@gazi.edu.tr; 5 Science and Technology Research and Application Center, Dicle University, 21280 Diyarbakır, Türkiye; mustafaabdullahyilmaz@gmail.com; 6 Department of Analytical Chemistry, Faculty of Pharmacy, Dicle University, 21280 Diyarbakir, Türkiye; 7 Department of Pharmaceutical Botany, Faculty of Pharmacy, Selcuk University, 42071 Konya, Türkiye; otugay@selcuk.edu.tr

**Keywords:** *Helichrysum plicatum* subsp. *plicatum*, varicose vein, in vitro enzyme inhibition activity, antioxidant activity, flavonoids, naringenin, apigenin, luteolin, LC-MS/MS

## Abstract

**Background:** Varicose veins and chronic venous insufficiency are chronic venous disorders involving abnormalities in the venous system. Inflammation, an increase in proteolytic enzymes, and free radicals are important factors that play a role in the varicose vein pathology. **Methods:** In this study, the antioxidant properties and inhibitor activities of 17 plant extracts used to treat varicose veins in traditional medicine were evaluated against varicose veins-related enzymes (hyaluronidase, elastase, collagenase, lipooxygenase, prolylendopeptidase, and xanthine oxidase). The most effective compounds responsible for the activity of the *Helichrysum plicatum* subsp. *plicatum* extract were isolated by open column chromatography techniques. The active compounds were determined to be naringenin, apigenin, and luteolin by spectroscopic methods. In the activity-guided isolation study, the xanthine oxidase enzyme inhibition method was used. **Results:** The fractions containing naringenin and apigenin (IC_50_ = 0.269 ± 0.009 µg/mL) and apigenin and luteolin (IC_50_ = 0.285 ± 0.019 µg/mL) compounds showed synergistic and strong effects against xanthine oxidase and were found to be as active as the positive control allopurinol (IC_50_ = 0.250 ± 0.006 µg/mL). In the LC-MS/MS analysis of the *Helichrysum plicatum* extract, quinic acid (22.649 mg compound/g extract), chlorogenic acid (14.573 mg/g extract), isoquercitrin (14.371 mg/g extract), cosmosin (9.885 mg/g extract), and astragalin (11.506 mg/g extract) were detected as the major components. Naringenin, apigenin, and luteolin were detected at concentrations of 1.457, 2.518, and 1.368 mg/g in the extract, respectively. **Conclusions:** In conclusion, it is predicted that the combination of naringenin, apigenin, and luteolin has a promising use as a conservative treatment option for diseases associated with varicose veins due to their synergistic effects with each other.

## 1. Introduction

People have long used plant-based medicines to treat a wide range of physical and mental ailments. Various tribes around the world utilise them as part of their traditional medicine. Medicinal and Aromatic Plants (MAPs) are becoming increasingly important, as they can be applied in the food, pharmaceutical, and beauty industries. The use of MAPs in everyday life highlights the necessity for future multidisciplinary research studies to provide scientific evidence of their benefits [1,2].

Chronic venous insufficiency (CVI) is often an underdiagnosed condition that diminishes patients’ quality of life and places a financial strain on healthcare resources [3]. Although CVI in the lower extremities can sometimes be asymptomatic, it frequently presents a wide clinical spectrum, ranging from cosmetic issues to serious symptoms [4]. Healthy valves and muscle pumps are essential to ensure that the blood in the venous system returns to the heart against gravity. When this system is insufficient, it leads to backward blood flow, blood stasis, and venous hypertension. Venous hypertension can trigger various events, including inflammation and venous valve insufficiency [5,6]. Inflammation and oxidative stress are contributing factors to both the development and progression of CVI. It is believed that abnormal venous flow triggers the inflammatory process in varicose veins. Lipoxygenase (LOX) enzyme inhibitors may be effective in treating varicose veins by suppressing the inflammatory response and preventing the formation of free radicals [7]. Previous research has indicated that prolylendopeptidase (POP) inhibitors enhance histological abnormalities in the venous wall while reducing the inflammatory process associated with CVI. Consequently, POP inhibitors are regarded as a new therapeutic target in the treatment of CVI [8]. Collagenase, hyaluronidase, and elastase enzymes damage venous structures when proteoglycan synthesis is low. It has been noted that the elastic lamina [9], collagen [10], and hyaluronic acid [11] content in the walls of varicose veins decreases [12]. Xanthine oxidase (XO) can catalyse the transfer of monovalent and divalent electrons to O_2_, producing two reactive oxygen species (ROS), superoxide anion (O_2_^•−^) and hydrogen peroxide (H_2_O_2_) [13]. Therefore, increased XO levels result in the release of ROS, exacerbating the pathology of varicose veins [14].

There are various techniques for treating superficial venous insufficiency. The treatment options for varicose veins include conservative methods (compression, lifestyle changes, and venoactive drugs) and surgical interventions (surgical ligation, stripping, microphlebectomy, sclerotherapy, and laser ablation) [6,15]. The selection of treatment methods depends on the nature of the varicose veins and the underlying physiological causes [16]. Medicinal plants and natural compounds represent one of the treatment strategies for CVI and varicose veins due to their biological activities, including anti-inflammatory effects, antioxidant properties, ability to increase vascular tone, ability to inhibit lysosomal enzymes, and anti-elastase properties [17,18]. Notably, coumarins, micronised purified flavonoid fractions, rutin derivatives (troxerutin and oxerutin), pycnogenol, anthocyanidins (delphinidin, cyanidin, and malvidin), proanthocyanidin from *Vitis vinifera* L. seed extracts, *Ruscus aculeatus* L., asiaticoside (*Centella asiatica* L.), *Mammea africana* Sabine extracts, fraxin, ginkgolide B (*Ginkgo biloba* L.), and saponosides (escin) play a significant role in the treatment of CVI [8,19].

In previous studies, various plants were used both externally and internally in the treatment of varicose veins in many different countries [19]. *Berberis vulgaris* L. [20], *Brassica oleracea* var. *acephala* L. [21], *Capsella bursa-pastoris* (L.) Medik. [22], *Capsicum annuum* L. [23], *Crataegus monogyna* Jacq. subsp. *Monogyna* [24], *Ecbalium elaterium* (L.) A.Rich. [25], *Helichrysum plicatum* DC. subsp. *plicatum* [26], *Lamium album* L. [27], *Lythrum salicaria* L. [28], *Plantago lanceolata* L. [29], *Plantago major* L. subsp. *major* [29], *Sambucus ebulus* L. [21], *Sesamum indicum* L. [30], and *Urtica dioica* L. [31] are utilised in the treatment of varicose veins in traditional medicine in Türkiye.

**Figure 1:**
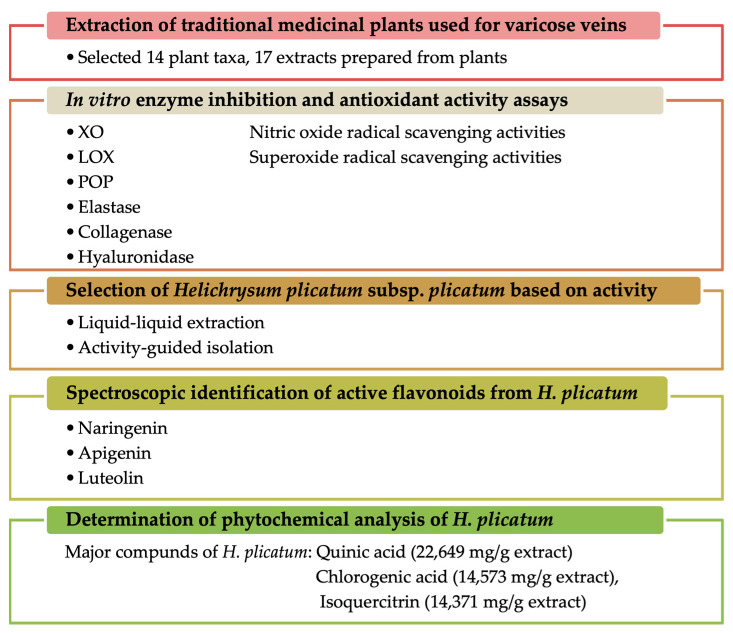
Summary of the experimental design and the main findings.

In this study, we aimed to elucidate the mechanism of action of plants that have been ethnopharmacologically used in the treatment of varicose veins, utilising various in vitro methods associated with varicose veins. The plant materials employed against varicose veins in traditional medicine were evaluated in terms of in vitro inhibition of the XO, elastase, collagenase, hyaluronidase, LOX, and POP enzymes, as well as antioxidant activities (including nitric oxide and superoxide anion radical-scavenging activities). The study also aimed to identify the compounds responsible for these activities through activity-guided fractionation. Furthermore, the phytochemical content of *H. plicatum* was quantitatively elucidated using LC-MS/MS. Thus, the effects of traditionally used plants were investigated through various mechanisms of action, and active compounds were isolated from the most effective plant, *Helichrysum plicatum* DC. subsp. *plicatum* (Figure 1).

## 2. Results

### 2.1. Results of Activity-Guided Isolation

**Figure 2:**
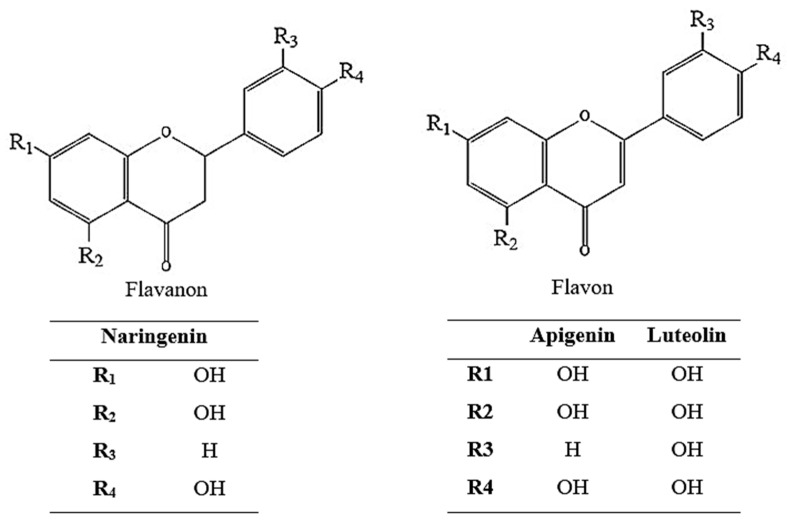
Chemical structure of naringenin, apigenin, and luteolin.

As a result of the activity-guided isolation studies, compounds 1, 2, and 3 were isolated from the EtOAc sub-extract. The isolated compounds were identified (Figure 2 and Table 1) using ^1^H-NMR, ^13^C-NMR, HMBC, COSY, and HMQC spectroscopic methods (Supplementary Material File S1). Furthermore, comparison of the spectral data of the compounds with spectra in the literature identified them as naringenin [32], apigenin [33], and luteolin [34].

### 2.2. LC-MS/MS Analysis Results

**Figure 3:**
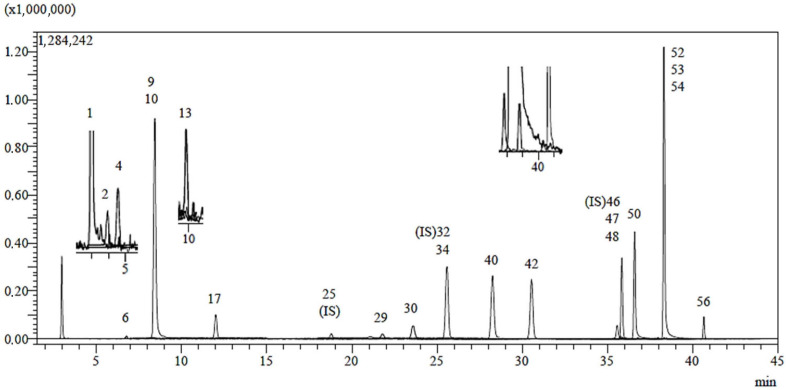
LC-MS/MS chromatogram of *H. plicatum* ethanolic extract.

In the phytochemical analysis conducted on the ethanolic extract of *H. plicatum* aerial parts, 53 phenolic compounds were analysed, and a total of 20 phenolic compounds were identified (Figure 3 and Table 2). Quinic acid (22.649 mg/g extract), chlorogenic acid (14.573 mg/g extract), isoquercitrin (14.371 mg/g extract), cosmosiin (9.885 mg/g extract), and astragalin (11.506 mg/g extract) were identified as the major components. Naringenin, apigenin, and luteolin, which were isolated through activity-guided isolation from the *H. plicatum* extract, were found at concentrations of 1.457 mg/g extract, 2.518 mg/g extract, and 1.368 mg/g extract, respectively. Additionally, benzoic acid, fumaric acid, gallic acid, protocatechuic acid, caffeic acid, protocatechuic aldehyde, salicylic acid, cynaroside, quercetin, kaempferol, amentoflavone, 1,5-dicaffeoylquinic acid, caffeic acid, and acacetin compounds were found in low amounts in the *H. plicatum* extract.

### 2.3. Results of Enzyme Inhibition Assays

The findings regarding the collagenase, hyaluronidase, XO, and LOX enzyme inhibition effects of the extracts are given in Table 3. None of the extracts were found to have elastase enzyme inhibitory activity.

**Figure 4:**
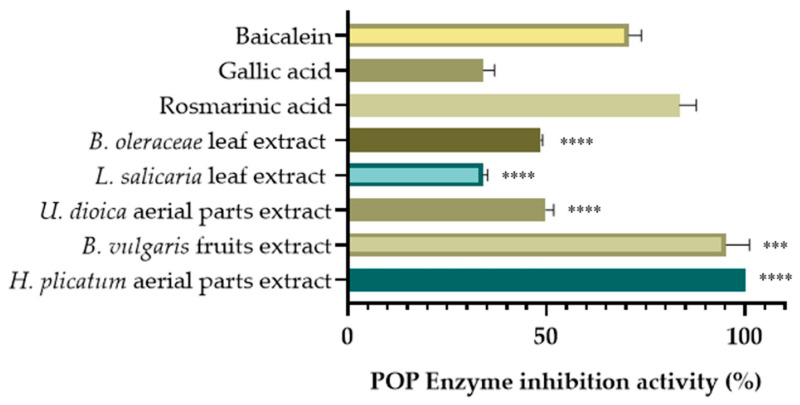
POP inhibitory activities of selected extracts. (*** *p* < 0.001, **** *p* < 0.0001).

Among the screened extracts, the extract of the *H. plicatum* aerial part (IC_50_ = 85.85 ± 0.91 µg/mL) exhibited high XO inhibitory activity, while the *L. salicaria* leaf (IC_50_ = 70.76 ± 3.34 µg/mL), B. vulgaris fruit (IC_50_ = 114.63 ± 5.86 µg/mL), and *B. oleraceae* leaf (IC_50_ = 124.85 ± 0.64 µg/mL) extracts demonstrated significant hyaluronidase inhibitory activity. The extract of the *U. dioica* aerial parts (45.82% ± 2.48) also displayed considerable LOX enzyme inhibitory activity and was evaluated for POP enzyme inhibitory activity at a final concentration of 100 µg/mL (Figure 4).

For the activity-guided isolation, the *H. plicatum* extract with the highest XO (IC_50_ = 85.85 ± 0.91 µg/mL) and POP inhibitory activities (99.99% ± 0.01) was selected. Based on the enzyme inhibitory activities of the sub-extracts (Table 4), the ethyl acetate (EtOAc) extract of *H. plicatum* was chosen for further isolation studies as it was effective against both hyaluronidase (76.28 ± 4.10%) and XO (IC_50_ = 28.06 ± 2.70 µg/mL). None of the sub-extracts exhibited anti-elastase activity. Due to the high XO inhibitory activity of *H. plicatum* and its EtOAc sub-extract, the activity of the fractions and pure compounds obtained from the activity-guided isolation study against the XO enzyme was assessed.

The inhibitory activities of the fractions obtained using Sephadex LH-20 (Sep. Fr.), silica gel (Si) and vacuum reverse-phase (RP1) silica gel column chromatography techniques against XO are presented in Table 5. The most active fractions isolated from the EtOAc sub-extract via Sephadex LH-20 column chromatography were Sep. Fr. 50–55 (IC_50_ = 9.18 ± 0.31 µg/mL) and Sep. Fr. 56–80 (IC_50_ = 3.82 ± 0.17 µg/mL). Among the fractions obtained from silica gel column chromatography for Sep. 50–55, the most active were Si. 1–4 and Si. 8–11. For the fractions acquired from reverse-phase silica gel column chromatography for Sep. 56–80, the most active fraction was RP 66–77. The Si. 1–4, Si. 8–11, and RP 66–77 fractions exhibited similar TLC profiles; consequently, the subsequent activity-guided isolation studies used these fractions.

**Figure 5:**
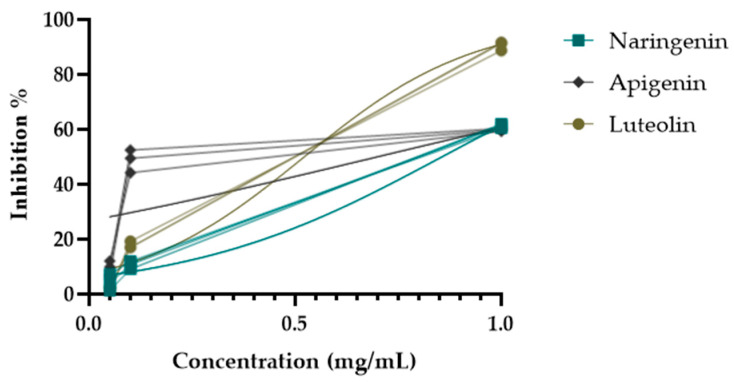
XO enzyme inhibitory activities of isolated compounds.

**Figure 6:**
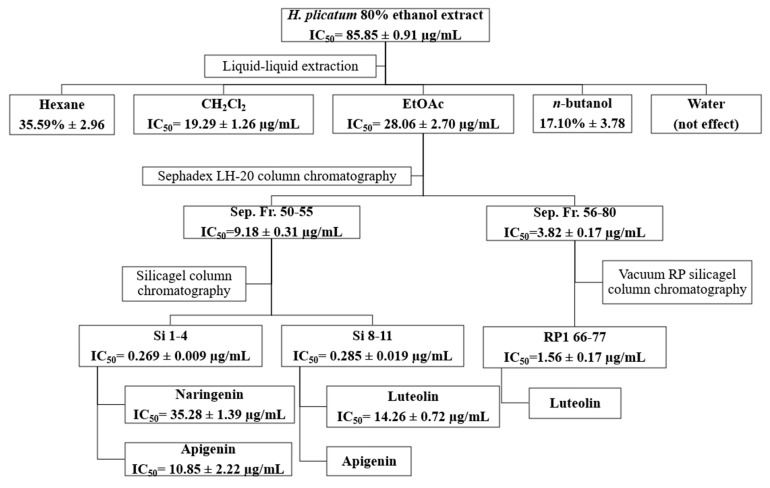
Isolation of the active compounds by activity-guided fractionation.

During the activity-guided isolation study of the *H. plicatum* extract, the inhibitory activity against XO increased. Apigenin (IC_50_ = 10.85 ± 2.22 µg/mL****) was identified as having a higher activity than luteolin (IC_50_ = 14.26 ± 0.72 µg/mL****) and naringenin (IC_50_ = 35.28 ± 1.39 µg/mL****) (Figure 5). Figure 6 provides a summary of the activity-guided isolation study results.

### 2.4. Results of Antioxidant Activity Assays

The nitric oxide and superoxide radical-scavenging activities of the 80% ethanol extracts of the plant materials are presented in Table 6. In terms of nitric oxide radical-scavenging activity, *H. plicatum* (IC_50_ = 152.70 ± 3.68 µg/mL) and *L. salicaria* (IC_50_ = 133.00 ± 6.79 µg/mL) extracts exhibited high antioxidant activity. For the determination of superoxide radical-scavenging activity, the experimental results were obtained using vitamin C as the standard. *L. salicaria* demonstrated the strongest effect (274.77 mg vit C equivalent/g extract).

## 3. Discussion

Approximately 150,000 new patients are diagnosed with CVI each year, and about USD 500 million is allocated to care for these patients [35]. The treatment of varicose veins and CVI is challenging because the cause remains unclear, and various factors contribute to the pathophysiology. Interventional treatment aimed at improving venous function is considered the first-line approach before other symptomatic treatment options are employed. If interventional treatment is not applicable or desired, or if symptoms persist following a therapeutic intervention, compression and pharmacological therapy may be used alone or in combination as symptom-based treatment options [36]. The most important group of pharmacological treatments is venoactive drugs [37,38]. The mechanism of action of venoactives is based on reducing capillary permeability, decreasing the release of inflammatory mediators, and enhancing venous tone. Generally, the application of flavonoids or flavonoid-rich fractions is beneficial in achieving therapeutic outcomes. Other secondary metabolites that positively affect vascular walls include triterpenic saponins. The mechanism of action of triterpenic saponins differs somewhat and includes the inhibition of certain enzymes responsible for degrading the structure of the vascular wall [5,39]. In venous pathophysiology, elevated venous pressure and changes in the flow force create an abnormal biomechanical environment in the vascular walls and valves. These biomechanical abnormalities lead to the premature release and activation of enzymes that induce endothelial dysfunction, degrade the extracellular matrix, and trigger a cascade of leukocyte infiltration and inflammation [40]. XO, hyaluronidase, elastase, collagenase, LOX, and POP are among the enzymes that create the abnormal conditions, such as inflammation, decreased resistance to venous pressure, increased vascular permeability, and oxidative stress, observed in CVI. Therefore, it is crucial to identify medicinal plants that can inhibit these enzymes for the treatment of varicose veins and CVI.

In our study, we first prepared 80% ethanolic extracts of plants used in traditional medicine for treating varicose veins to obtain various secondary metabolites from different plant parts, and conducted in vitro activity tests. When the enzyme inhibition results of the extracts were evaluated, the extract of *H. plicatum*, which demonstrated the highest inhibitory activity against POP (99.99% ± 0.01) and XO (IC_50_ = 85.85 ± 0.91 µg/mL), was selected for activity-guided isolation studies. *H. plicatum* has not previously been studied for the inhibition of enzymes associated with varicose veins. When the activity of the other plant extracts was assessed, it was found that the *B. vulgaris* fruit extract (95.24% ± 5.84) exhibited significant inhibitory activity against the POP enzyme. It is predicted that this effect may be due to berberine [41], which is known to be the major component of *B. vulgaris*. Studies have confirmed that berberine (IC_50_ = 142.0 ± 21.5 µM) is a potent POP inhibitor [42,43]. The extract from *U. dioica* (45.82% ± 2.48), which had the highest inhibitory activity against the LOX enzyme, has been extensively studied for its inflammation-reducing properties. In one study, after *U. dioica* was administered orally to experimental animals, it inhibited rat paw oedema similarly to indomethacin. The anti-inflammatory activity of *U. dioica* is attributed to the inhibition of cyclooxygenase, LOX, and cytokine production [44]. The extract from *S. indicum* seeds exhibited moderate inhibitory activity against the LOX enzyme (IC_50_ = 41.07 ± 3.77). Research has shown that sesamol, a lignan structure found in *S. indicum*, possesses strong LOX inhibitory properties that act in a dose-dependent manner (IC_50_ = 51.84 μM) [45]. Our results indicate that the *L. salicaria* leaf extract (IC_50_ = 70.76 ± 3.34 μg/mL) displayed the highest inhibitory activity against the hyaluronidase enzyme. In another study, it was demonstrated that the aerial parts of *L. salicaria* inhibited hyaluronidase activity in a dose-dependent manner with an IC_50_ of 10.1 ± 1.2 μg/mL and induced 94.4 ± 0.6% inhibition at a concentration of 20 μg/mL [46]. Further investigations revealed that *L. salicaria* achieved 64.9% (IC_50_ = 8.1 ± 0.8 μg/mL) inhibition of the hyaluronidase enzyme at a concentration of 10 μg/mL [47]. However, the NO radical- (IC_50_ = 133.00 ± 6.79 μg/mL) and superoxide anion-scavenging (274.77 vit C mg/extract g) activities of *L. salicaria* were the highest among our extracts. In one study, the IC_50_ values of the *L. salicaria* leaf extracts prepared using various methods showed an NO radical-scavenging activity between 0.73 ± 0.03 mg/mL and 1.41 ± 0.07 mg/mL. This activity was found to be weaker than that of quercetin (IC_50_ = 0.16 ± 0.01 mg/mL), which was used as the positive control [48]. In our study, it was observed that the *L. salicaria* extract demonstrated a weaker effect than vitamin C (IC_50_ = 25.28 ± 2.98 µg/mL), which was also used as a positive control. It can be concluded that this study provided results similar to those of previous studies.

The sub-extracts were prepared with different polarities using the *H. plicatum* ethanolic extract. The activity-guided isolation study used the EtOAc sub-extract, which was the most active against XO (IC_50_ = 28.06 ± 2.70 µg/mL) and hyaluronidase (76.28% ± 4.10). Due to the inhibitory activity of the *H. plicatum* extract against XO, this enzyme was selected as the determinant in the activity-guided isolation study. Eight fractions were obtained through Sephadex LH-20 column chromatography, with the active fractions being Sep. Fr. 50–55 (IC_50_ = 9.18 ± 0.31 µg/mL) and Sep. Fr. 56–80 (IC_50_ =3.82 ± 0.17 µg/mL). An evaluation of the TLC profiles of the Sep. Fr. 50–55 and Sep. Fr. 56–80 fractions showed that they contained similar compounds, which were targeted for the next step of the isolation process. Sep. Fr. 50–55 was separated using the silica gel column chromatography technique, while Sep. Fr. 56–80 was separated using vacuum reverse-phase silica gel column chromatography. In the isolation studies, the preparative TLC method was also employed, resulting in the isolation of compound **1**, compound **2**, and compound **3**. The isolated compounds were identified by NMR to be naringenin, apigenin, and luteolin, respectively. Although the phytochemical content of *H. plicatum* has been elucidated through advanced chromatographic techniques, no isolation studies focused on biological activity have been conducted. This study marks the first time that three compounds predicted to be active were isolated from *H. plicatum*. Phytochemical studies of *H. plicatum* have confirmed the presence of compounds such as apigenin, naringenin, luteolin, kaempferol, and their glycosides, quercetin, dicaffeoylquinic acid, isoquercitrin, β-sitosterol, nonacosanoic acid, astragalin, *β*-sitosterol-3-*O*-*β*-D-glucoiranoside, helicrhysin A, helicrhysin B, isosalipurposide, chlorogenic acid, and chalcone derivatives [49,50,51]. In our study, we detected quinic acid, benzoic acid, fumaric acid, gallic acid, protocatechuic acid, chlorogenic acid, caffeic acid, protocatechuic aldehyde, salicylic acid, cynaroside, isoquercitrin, cosmosiin, astragalin, quercetin, naringenin, luteolin, kaempferol, apigenin, amentoflavone, and acacetin compounds in the *H. plicatum* extracts. These results are similar to those of other phytochemical studies.

During the activity-guided isolation study process starting from *H. plicatum*, the inhibitory activity against XO increased until the extract was reduced to the pure compounds. Si. 1–4 fractions (IC_50_ = 0.269 ± 0.009 µg/mL), naringenin, and apigenin were identified as the major constituents of the TLC profile. Si. 8–11 (IC_50_ = 0.285 ± 0.019 µg/mL), where apigenin and luteolin were the primary components in the TLC profile and exhibited a synergistic effect with one another, was found to be as effective as the positive control allopurinol (IC_50_ = 0.250 ± 0.006 µg/mL). When the inhibitory activities of the isolated compounds naringenin (IC_50_ = 35.28 ± 1.39 µg/mL), apigenin (IC_50_ = 10.85 ± 2.22 µg/mL), and luteolin (IC_50_ = 14.26 ± 0.72 µg/mL) against the XO enzyme were evaluated individually, the effect was weaker (Figure 6).

In other studies, naringenin demonstrated moderate inhibitory activity against XO [52,53]. Additionally, another study found that naringenin (IC_50_ = 4.57 μmol/L) was as active as the positive control allopurinol (IC_50_ = 4.05 μmol/L) [54]. One study revealed that apigenin was a strong competitive inhibitor of XO with an IC_50_ of 3.57 μM. The phenolic group of apigenin extends to the vicinity of the hydrophobic cavity, the active site of XO, through hydrogen bonds and electrostatic interactions [55]. Among the flavonoids isolated from *Blumea balsamifera* L., luteolin (IC_50_ = 2.38 ± 0.01 µM) exhibited the highest XO inhibitory activity [56]. Furthermore, another study found that luteolin (IC_50_ = 0.96 µM) displayed the highest activity after kaempferol, quercetin, and isorhamnetin [57].

Regarding the structure–activity relationship of flavonoids, it has been reported that the double bond at C-2 and C-3, along with the OH groups at C-5 and C-7, play an important role in inhibiting XO [58,59]. Our study observed that naringenin, which has lower XO-inhibiting activity compared to other flavonoids, may have a reduced effect due to the saturation of the double bond at C-2 and C-3. Previous studies have shown that the inhibitory activity against and affinity for XO decrease in the presence of the OH group at the C-3′ position in the B ring [59]. In this study, luteolin may exhibit lower XO-inhibiting activity than apigenin due to the OH group at C-3′.

**Figure 7:**
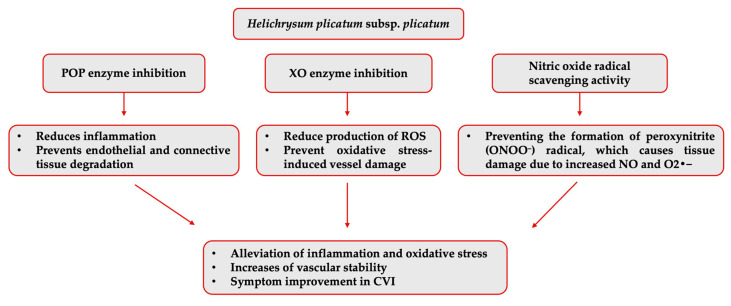
Potential mechanism of action of *H. plicatum* extract against varicose veins.

The isolated compounds were not individually evaluated regarding their inhibitory activities against the other varicose vein-related enzymes. However, another enzyme that *H. plicatum* (99.99% ± 0.01) has displayed high inhibitory activity against is POP. Activity studies have found that naringenin [60] did not exhibit inhibitory activity against the POP enzyme, and luteolin showed high inhibitory activity (IC_50_ = 0.17 ppm); there was no data for apigenin [61]. Luteolin may contribute to the high activity of *H. plicatum*. However, previous structure–activity studies suggested that quinic acid and chlorogenic acid, which are the predominant components in *H. plicatum* extracts, may be responsible for the POP inhibitory activity due to the presence of the pyrogallol group [62]. This study determined that naringenin, apigenin, and luteolin, as the potential active compounds in the *H. plicatum* extract that can treat varicose veins, may help reduce oxidative stress by supporting antioxidant mechanisms in CVI due to their strong ability to inhibit the XO enzyme when combined (Figure 7).

## 4. Materials and Methods

### 4.1. Plant Materials and Extraction Methods

Information on the plant species screened in this study is presented in Table 7. *H. plicatum* was collected from Osmaniye province, while the other plant taxa were sourced from Konya province. The collected plant samples were identified by Prof. Dr. Osman Tugay. Herbarium samples have been preserved in the herbarium of the Faculty of Science, Selçuk University.

All plant samples were macerated using 80% ethanol at room temperature for 5 days. Subsequently, filter paper was used to filter the samples, and the remaining filtrate was evaporated to dryness in a rotary evaporator (Büchi, Flawil, Switzerland). The extracts were weighed and dissolved in dimethyl sulfoxide (DMSO) to reach a concentration of 2 mg/mL, which was used in the in vitro experiments.

### 4.2. Activity-Guided Isolation Study

The ethanol extracts of *H. plicatum* (30 g) were dissolved in 90% methanol (MeOH) and subjected to liquid–liquid fractionation using hexane, dichloromethane (CH_2_Cl_2_), EtOAc, *n*-butanol, and water. The organic phases were evaporated to dryness, yielding five fractions along with the remaining aqueous phase. Finally, the aqueous phase was lyophilised.

#### 4.2.1. Fractionation of EtOAc Extract

These fractions were subsequently screened for their inhibitory activities against varicose veins-related enzymes. The most active EtOAc (4.9966 g) fraction was fractionated using Sephadex LH-20 column chromatography (3 cm × 60 cm). MeOH served as the mobile phase for the separation. The profiles that exhibited similarities in their TLC profiles were combined, yielding a total of eight fractions (Sep. Fr.). The XO enzyme inhibitory activities of these fractions (Sep. Fr.) were tested.

#### 4.2.2. Fractionation of Sep. Fr. 50–55

The active fraction Sep. Fr. 50–55 was separated using the silica gel column chromatography technique [mobile phase: chloroform CHCl_3_/MeOH/water at ratios of 90:10:1, 80:20:2, 70:30:3, 61:32:7, and 50:5:5]. Compounds 1 and 2 were isolated from the Si 1–4 fraction using the preparative (prep) TLC method [stationary phase: silica gel; mobile phase: CHCl_3_/MeOH/water (90:10:1)].

#### 4.2.3. Fractionation of Sep. Fr. 56–80

Sep. Fr. 56–80 was separated into four fractions using the vacuum reverse-phase silica gel column chromatography technique [mobile phase: water/MeOH at ratios of 100:0, 98:2, 93:7, 80:20, 75:25, 70:30, 50:50, 25:75, and 0:100]. The most active RP1 66–77 fraction was isolated from compound **3** by employing the vacuum reverse-phase silica gel column chromatography technique [mobile phase: water/MeOH at ratios of 85:15, 82:18, 79:21, 75:25, 72:28, 70:30, 68:32, 65:35, 60:40, 57:43, 55:45, and 50:50].

### 4.3. LC-MS/MS Analysis Methods

The LC-MS/MS analysis was performed using a Shimadzu Nexera model ultrahigh-performance liquid chromatography (UHPLC) system, coupled with a tandem mass spectrometer, to quantitatively evaluate 53 phytochemicals. The conditions of the chromatographic analysis were the same as those used in previous studies [63,64].

### 4.4. Microtiter Assays for Enzyme Inhibition

The enzyme inhibition assays conducted in this study included elastase, collagenase, hyaluronidase, XO, and prolyl POP inhibition and were performed using an ELISA microplate reader (Spectramax i3x microplate reader with Softmax^®^ Pro Software for Windows 10; Molecular Devices, San Jose, USA). Elastase inhibition by the extracts was assessed using a modified spectrophotometric method based on Kraunsoe et al. (1996) [65] and Lee et al. (2009) [66]. Collagenase inhibition was evaluated using the modified spectrophotometric method by Barrantes and Guinea 2003 [67], which was originally developed by Wart and Steinbrink (1981) [68]. The LOX inhibitory activities of the extracts were determined following the method of Chung et al. (2009), with minor modifications [69]. XO inhibition was measured spectrophotometrically by monitoring the absorbance at 295 nm from uric acid formed during an enzymatic reaction [70]. Inhibition of the POP enzyme was conducted on the five extracts that exhibited the highest activity for the other enzymes. To determine POP enzyme inhibitory activity, a Fluorogenic Prolyl OligoPeptidase (POP) test kit (catalogue number: 80106) from BPS Bioscience Inc. (San Diego, CA, USA) was utilised.

### 4.5. Antioxidant Activity Assays

The determination of the superoxide radical (O_2_^•−^)-scavenging effect was conducted using the method described by Duh et al. [71,72]. To assess the nitric oxide (NO)-scavenging effect, the method of Sreejayan and Rao was utilised with some modifications [73].

### 4.6. Statistical Analysis

For the activity assays, the test groups were compared with the positive control using one-way ANOVA followed by Dunnett’s multiple comparison test and the results were statistically analysed (GraphPad Prism 8.0). *p* values ≤ 0.05 were considered statistically significant.

## 5. Conclusions

In conclusion, an activity-guided isolation study was conducted and the *H. plicatum* extract demonstrated the highest activity in in vitro enzyme inhibition assays. Naringenin, apigenin, and luteolin were identified as the most active compounds. The fact that these isolated compounds belong to the flavonoid class, which includes venoactive drugs used to treat CVI disease, underscores the importance of flavonoids in this condition. This study determined that naringenin, apigenin, and luteolin may help reduce oxidative stress by bolstering the antioxidant mechanisms in CVI due to their ability to inhibit the XO enzyme. Furthermore, the observation that the naringenin–apigenin and apigenin–luteolin fractions displayed XO inhibitory activity as robust as that of allopurinol suggests that these compounds might have synergistic effects, and their combined application may yield more effective results for CVI. Our research into active extracts and isolated compounds using the human endothelial cell line (HUVEC) is ongoing. We are currently conducting a cell culture test using HUVECs and chemically inducing hypoxia to mimic the conditions associated with varicose veins. We plan to perform in vivo experiments based on the results obtained from cell culture and in silico drug-likeness parameters, as well as ADME profile analysis of active and major compounds in future studies.

## Figures and Tables

**Table 1:** ^1^H and ^13^C NMR spectrum results for naringenin, apigenin, and luteolin.

Carbon	Naringenin		Apigenin		Luteolin	
	δC	δH	δC	δH	δC	δH
C-2	78.87	5.44 (d, *J* = 12.5 Hz)	164.11	-	165.71	-
C-3	42.44	3.26 (m), 2.72 (dd, *J* = *, 17.1)	99.45	6.17 (br s)	102.40	6.57 (s)
C-4	196.63	-	182.12	-	182.9	-
C-5	163.95	12.15 (s)	161.91	12.96 (s)	164.42	13.03 (s)
C-6	96.31	5.87 (br s)	94.50	6.46 (d, *J* = 2.5 Hz)	99.82	6.12 (br s)
C-7	165.62	12.15 (s)	165.27	12.96 (s)	166.13	13.03 (s)
C-8	95.49	5.87 (br s)	103.22	6.77 (d, *J* = 2.5 Hz)	94.41	6.38 (br s)
C-9	163.39	-	103.92	-	161.84	-
C-10	102.14	-	157.82	-	103.43	-
C-1′	129.96	-	121.57	-	120.61	-
C-2′	128.79	7.31 (d, *J* = 8.3 Hz)	128.90	7.92 (d, *J* = 8 Hz)	113.31	7.32 (m)
C-3′	115.62	6.79 (d, *J* = 8.1 Hz)	116.45	6.93 (d, *J* = 8 Hz)	146.81	-
C-4′	158.20	-	161.79	10.78 (s)	151.96	-
C-5′	115.62	6.79 (d, *J* = 8.1 Hz)	116.45	6.93 (d, *J* = 8 Hz)	116.40	6.82 (d, *J* = 7.2 Hz)
C-6′	128.79	7.31 (d, *J* = 8.3 Hz)	128.90	7.92 (d, *J* = 8 Hz)	119.32	7.32 m

* J value could not be determined due to solvent overlap.

**Table 2:** Identification and quantification of phenolic compounds in *H. plicatum* ethanolic extract.

No.	Analyte	R.T. ^a^	M.I. (m/z) ^b^	Ion. Mode	Equation	*r* ^2 c^	*LOD/LOQ* (µg/L) ^d^	mg/g Extract
1	Quinic acid	3.0	190.8	Neg	*y* = −0.0129989 + 2.97989×	0.996	25.7/33.3	22.649
2	Fumaric aid	3.9	115.2	Neg	*y* = −0.0817862 + 1.03467x	0.995	135.7/167.9	0.656
4	Gallic acid	4.4	168.8	Neg	*y* = 0.0547697 + 20.8152x	0.999	13.2/17.0	0.021
6	Protocatechuic acid	6.8	152.8	Neg	*y* = 0.211373 + 12.8622x	0.957	21.9/38.6	0.236
9	Chlorogenic acid	8.4	353.0	Neg	*y* = 0.289983 + 36.3926x	0.995	13.1/17.6	14.573
10	Protocatechuic aldehyde	8.5	137.2	Neg	*y* = 0.257085 + 25.4657x	0.996	15.4/22.2	0.069
13	1,5-dicaffeoylquinic acid	9.8	515.0	Neg	*y* = −0.0164044 + 16.6535x	0.999	5.8/9.4	0.055
17	Caffeic acid	12.1	179.0	Neg	*y* = 0.120319 + 95.4610x	0.999	7.7/9.5	0.544
29	Salicylic acid	21.8	137.2	Neg	*y* = 0.239287 + 153.659x	0.999	6.0/8.3	0.032
30	Cyranoside	23.7	447.0	Neg	*y* = 0.280246 + 6.13360x	0.997	12.1/16.0	1.634
34	Isoquercitrin	25.6	463.0	Neg	*y* = −0.111120 + 4.10546x	0.998	8.7/13.5	14.371
40	Cosmosiin	28.2	431.0	Neg	*y* = −0.708662 + 8.62498x	0.998	6.3/9.2	9.885
42	Astragalin	30.4	447.0	Neg	*y* = 0.00825333 + 3.51189x	0.999	6.6/8.2	11.506
47	Quercetin	35.7	301.0	Neg	*y* = 0.00597342 + 3.39417x	0.999	15.5/19.0	0.319
48	Naringenin	35.9	270.9	Neg	*y* = −0.00393403 + 14.6424x	0.999	2.6/3.9	1.457
50	Luteolin	36.7	284.8	Neg	*y* = −0.0541723 + 30.7422x	0.999	2.6/4.1	1.368
52	Kaempferol	37.9	285.0	Neg	*y* = −0.00459557 + 3.13754x	0.999	10.2/15.4	0.091
53	Apigenin	38.2	268.8	Neg	*y* = 0.119018 + 34.8730x	0.998	1.3/2.0	2.518
54	Amentoflavone	39.7	537.0	Neg	*y* = 0.727280 + 33.3658x	0.992	2.8/5.1	0.003
56	Acacetin	40.7	283.0	Neg	*y* = −0.559818 + 163.062x	0.997	1.5/2.5	0.005

^a^ R.T.: retention time; ^b^ MI (*m*/*z):* molecular ions of the standard analytes (*m*/*z* ratio); ^c^ *r*^2^: coefficient of determination; ^d^ *LOD*/*LOQ* (µg/L): limit of detection/quantification.

**Table 3:** Enzyme inhibitory activities of plants used in the treatment of varicose veins.

		Enzyme Inhibition (% Inhibition ± S.D ^a^)			
Samples	Parts	XO 100 µg/mL ^b^	LOX 100 µg/mL ^b^	Collagenase 133.33 µg/mL ^b^	Hyaluronidase 166.66 µg/mL ^b^
*Berberis vulgaris*	Flos	26.52 ± 5.35 ****	- ^c^	-	59.12 **±** 5.60 ^d^ ****
	Fruit	24.13 ± 0.66 ****	-	29.16 ± 4.24 ****	70.10 ± 4.96 **** (IC_50_ = 114.63 ± 5.86 µg/mL)
*Brassica oleraceae* var. *acephala*	Leaf	7.89 ± 1.92 ****	-	-	75.60 ± 10.68 ** (IC_50_ = 124.85 ± 0.64 µg/mL)
*Capsella bursa pastoris*	Aerial part	16.74 ± 5.99 ****	16.07 ± 3.34 ****	-	42.02 ± 10.02 ****
*Lamium album*	Aerial part	13.35 ± 3.06 ****	-	-	7.74 ± 2.11 ****
*Plantago major*	Leaf	28.57 ± 2.27 ****	-	-	-
*Urtica dioica*	Aerial part	21.16 ± 2.26 ****	45.82 ± 2.48 ****	-	-
*Capsicum annuum*	Fruit	10.81 ± 5.60 ****	20.21 ± 1.80 ****	-	42.05 ±1.54 ****
*Helychrysum plicatum* subsp. *plicatum*	Aerial part	60.15 ± 0.65 **** (IC_50_ = 85.85 ± 0.91 µg/mL)	-	2.12 ± 0.44 ****	47.29 ± 3.12 ****
*Sesamum indicum*	Seed	17.20 ± 1.93 ****	41.07 ± 3.77 ****	-	39.05 ± 3.95 ****
*Sambucus ebulus*	Flos	20.80 ± 0.34 ****	-	-	41.80 ± 4.19 ****
	Leaf	23.32 ± 3.74 ****	-	-	64.56 ± 4.94 **** (IC_50_ = 139.55 ± 5.48 µg/mL)
*Crataegus monogyna*	Leaf	31.86 ± 3.91	-	-	-
	Fruit	10.78 ± 4.13	-	-	-
*Lythrum salicaria*	Leaf	27.64 ± 2.51 ****	-	-	84.60 ± 3.76 (IC_50_ = 70.76 ± 3.34 µg/mL)
*Ecballium elaterium*	Fruit	12.77 ± 1.57 ****	-	-	-
*Plantago lanceolata*	Leaf	31.17 ± 1.71 ****	-	-	-
References (1 mg/mL)		91.58 ± 1.30 ^e^ (IC_50_ = 0.250 ± 0.006 µg/mL)	60.10 ± 2.39 ^f^ (IC_50_ = 41.84 ± 1.35)	87.39 ± 2.85 ^g^ (IC_50_ = 27.95 ± 0.3 µg/mL)	92.51 ± 3.53 ^h^ (IC_50_ = 2.31 ± 0.73 µg/mL)

^a^ Standard deviation, ^b^ final concentration, ^c^ no activity, ^d^ inactive when the dose was reduced, IC_50_ could not be calculated, ^e^ allopurinol 0.5 mg/mL, ^f^ baicalein, ^g^ 1,10-phenanthroline, ^h^ tannic acid, **p < 0.01, **** *p* < 0.0001.

**Table 4:** Enzyme inhibitory activities of sub-extracts obtained from *H. plicatum* extract.

	Enzyme Inhibition (% Inhibition ± S.D ^a^)			
Sub-Extract	XO 100 µg/mL ^b^	LOX 100 µg/mL ^b^	Collagenase 133.33 µg/mL ^b^	Hyaluronidase 166.66 µg/mL ^b^
*n*-hexane	35.59 ± 2.96 ****	39.87 ± 1.17 ****	- ^c^	79.32 ± 3.21 ^d^ **
Dichloromethane	74.80 ± 1.63 **** (IC_50_ = 19.29 ± 1.26 µg/mL)	2.16 ± 0.61 ****	11.37 ± 2.33	-
EtOAc	70.44 ± 4.32 **** (IC_50_ = 28.06 ± 2.70 µg/mL)	-	-	76.28 ± 4.10 ^d^ ***
*n*-butanol	17.10 ± 3.78 ****	-	-	58.41 ± 6.27 ^d^ ****
Water	-	-	-	-
References (1 mg/mL)	91.58 ± 1.30 ^e^ (IC_50_ = 0.250 ± 0.006 µg/mL)	60.10 ± 2.39 ^f^ (IC_50_ = 41.84 ± 1.35)	87.39 ± 2.85 ^g^ (IC_50_ = 27.95 ± 0.37 µg/mL)	92.51 ± 3.53 ^h^ (IC_50_ = 2.31 ± 0.73 µg/mL)

^a^ Standard deviation, ^b^ final concentration, ^c^ no activity, ^d^ inactive when the dose was reduced, IC_50_ could not be calculated, ^e^ allopurinol 0.5 mg/mL, ^f^ baicalein, ^g^ 1,10-phenanthroline, ^h^ tannic acid, ** *p* < 0.01, *** *p* < 0.001, **** *p* < 0.0001.

**Table 5:** XO enzyme inhibitory activities of fractions obtained by various column chromatography methods from *H. plicatum* EtOAc sub-extract.

Fraction		XO Enzyme Inhibition (% Inhibition ± S.D. ^a^) 100 µg/mL ^b^
Fractions from Sephadex LH-20 column chromatography	Sep. Fr. 1–7	27.62 ± 2.21 ****
	Sep. Fr. 8–14	10.34 ± 1.71 ****
	Sep. Fr. 15–22	11.96 ± 2.58 ****
	Sep. Fr. 23–28	22.73 ± 1.49 ****
	Sep. Fr. 29–39	38.73 ± 3.07 ****
	Sep. Fr. 40–49	62.97 ± 5.74 (IC_50_ = 49.39 ± 1.17 µg/mL) ****
	Sep. Fr. 50–55	89.98 ± 2.44 (IC_50_ = 9.18 ± 0.31 µg/mL)
	Sep. Fr. 56–80	90.54 ± 2.14 (IC_50_ = 3.82 ± 0.17 µg/mL)
Fractions from silica gel column chromatography	Si 1–4	92.67 ± 1.60 ^c^ (IC_50_ = 0.269 ± 0.009 µg/mL)
	Si 5	77.95 ± 3.79 ^d^ (IC_50_ = 0.421 ± 0.046 µg/mL) ****
	Si 6–7	97.63 ± 0.13 (IC_50_ = 1.342 ± 0.255 µg/mL) *
	Si 8–11	93.83 ± 3.18 ^c^ (IC_50_ = 0.285 ± 0.019 µg/mL)
	Si 12–25	96.72 ± 1.22 (IC_50_ = 3.165 ± 0.440 µg/mL)
	Si 26–29	83.93 ± 3.59 ^e^ (IC_50_ = 3.978 ± 0.293 µg/mL) **
	Si 30–33	82.82 ± 4.17 ^e^ (IC_50_ = 4.866 ± 0.205 µg/mL) ***
	Si 35–37	93.46 ± 2.72 ^c^ (IC_50_ = 5.895 ± 0.079 µg/mL)
	Si 38–42	13.86 ± 0.28 ****
	Si 43–46	76.02 ± 1.66 (IC_50_ = 33.868 ± 3.760 µg/mL) ****
	Si 47–51	82.83 ± 2.50 (IC_50_ = 6.955 ± 0.496 µg/mL) ***
	Si 52–58	84.64 ± 3.93 (IC_50_ = 34.035 ± 1.558 µg/mL) *
	Si 59–60	76.92 ± 4.38 (IC_50_ = 54.013 ± 2.826 µg/mL) ****
Fractions from vacuum reverse-phase silica gel column chromatography	RP1 21–27	71.44 ± 0.43 (IC_50_ = 49.72 ± 0.39 µg/mL) ****
	RP1 37–39	72.56 ± 3.29(IC_50_ = 37.02 ± 1.76 µg/mL) ****
	RP1 51–55	92.11 ± 2.00 (IC_50_ = 2.49 ± 2.49 µg/mL)
	RP1 66–77	95.46 ± 0.07 (IC_50_ = 1.56 ± 0.17 µg/mL) *
Reference	Allopurinol ^c^	91.58 ± 1.30 (0.250 ± 0.006 µg/mL)

^a^ Standard deviation, ^b^ final concentration, ^c^ 25 µg/mL, ^d^ 5 µg/mL, ^e^ 50 µg/mL, * *p* < 0.1, ** *p* < 0.01, *** *p* < 0.001, **** *p* < 0.0001.

**Table 6:** Antioxidant activities of plants employed in the treatment of varicose veins in traditional medicine.

Sample	Part	Nitric Oxide Radical-Scavenging Activity (% Inhibition ± S.D. ^a^) 2 mg/mL ^b^	Superoxide Radical-Scavenging Activity (Vitamin C Equivalent) 2 mg/mL ^b^
*Berberis vulgaris*	Flower	75.95 ± 0.17 **** (IC_50_ = 225.50 ± 6.79 µg/mL)	39.76
	Fruit	67.88 ± 7.27 **** (IC_50_ = 326.70 ± 4.10 µg/mL)	48.33
*Brassica oleraceae* var. *acephala*	Leaf	44.52 ± 1.04 ****	0.003
*Capsella bursa pastoris*	Aerial part	53.15 ± 3.51 **** (IC_50_ = 433.25 ± 11.53 µg/mL)	1.3
*Lamium album*	Aerial part	70.72 ± 0.43 **** (IC_50_ = 315.00 ± 5.52 µg/mL)	3.56
*Plantago major*	Leaf	79.31 ± 0.86 **** (IC_50_ = 208.30 ± 3.68 µg/mL)	15.80
*Urtica dioica*	Aerial part	47.18 ± 1.28 ****	1.97
*Capsicum annuum*	Fruit	36.04 ± 0.95 ****	0.19
*Helychrysum plicatum* subsp. *plicatum*	Aerial part	82.66 ± 0.36 ** (IC_50_ = 152.70 ± 3.68 µg/mL)	73.38
*Sesamum indicum*	Seed	51.90 ± 0.00 **** (IC_50_ = 488.73 ± 0.81 µg/mL)	0.17
*Crataegus monogyna* subsp. *monogyna*	Fruit	53.27 ± 4.58.**** (IC_50_ = 499.80 ± 0.14 µg/mL)	4,54
	Leaf	81.30 ± 0.20 *** (IC_50_ = 165.05 ± 0.07 µg/mL)	80.09
*Sambucus ebulus*	Flower	74.07 ± 1.40 **** (IC_50_ = 270.70 ± 1.98 µg/mL)	3.29
	Leaf	72.54 ± 1.19 **** (IC_50_ = 312.70 ± 4.68 µg/mL)	4.96
*Lythrum salicaria*	Leaf	86.58 ± 0.55 (IC_50_ = 133.00 ± 6.79 µg/mL)	274.77
*Ecballium elaterium*	Fruit	59.86 ± 1.88 **** (IC_50_ = 432.40 ± 0.57 µg/mL)	0.08
*Plantago lanceolata*	Leaf	78.91 ± 3.39 **** (IC_50_ = 260.40 ± 1.70 µg/mL)	6.58
Vitamin C (1 mg/mL)		92.72 ± 0.10 (IC_50_ = 25.28 ± 2.98 µg/mL)	-

^a^ Standard deviation, ^b^ stock concentration, ** *p* < 0.01, *** *p* < 0.001, **** *p* < 0.0001.

**Table 7:** The extraction yields of plant materials and their applications in the treatment of varicose veins in traditional medicine.

Plant Material	Part	Yield% (*w*/*w*)	Family	Region	Preparation Method	Usage
*Berberis vulgaris* L.	Fruit	25.58	Berberidaceae	Cappadocia	Decoction	Oral [20]
	Flowers	25.74				
*Brassica oleraceae* var. *acephala*	Leaf	40.88	Brassicaceae	Samsun	Decoction	Topical [21]
*Capsella bursa pastoris* (L.) Medik.	Aerial part	8.00	Brassicaceae	Hatay	Decoction	Oral [22]
*Capsicum annuum* L.	Fruit	31.63	Solanaceae	Ankara	Boiled with bran flour	- * [23]
*Crataegus monogyna* Jacq. subsp. *monogyna*	Fruit	20.10	Rosaceae	Erzincan	-	- [24]
	Leaf	25.73				
*Ecballium elaterium* (L.) A.Rich.	Fruit	15.03	Cucurbitaceae	Çanakkale	-	- [25]
*Helichrysum plicatum* DC. subsp. *plicatum*	Aerial part	10.84	Asteraceae	Gümüşhane	-	- [26]
*Lamium album* L.	Aerial part	11.35	Lamiaceae	Adıyaman	-	Topical [27]
*Lythrum salicaria* L.	Leaf	11.54	Lythraceae	-	Decoction	Topical [28]
*Plantago lanceolata* L.	Leaf	11.83	Plantaginaceae	Balıkesir	Infusion	Topical [29]
*Plantago major* subsp. *major* L.	Leaf	14.08	Plantaginaceae	Balıkesir	Infusion	Topical [29]
*Sambucus ebulus* L.	Flowers	38.06	Viburnaceae	Samsun	-	Topical [21]
	Leaf	18.92				
*Sesamum indicum* L.	Seed (fixed oil)	7.31	Pedaliaceae	Mersin	-	- [30]
*Urtica dioica* L.	Aerial part	7.09	Urticaceae	Kırklareli	Decoction	Topical [31]

* The information was not included in the manuscript.

## Data Availability

The original contributions presented in this study are included in the article/Supplementary Material. Further inquiries can be directed to the corresponding author.

## Supplementary Materials

The following supporting information can be downloaded at: https://www.mdpi.com/article/10.3390/ph18060926/s1, Figure S1: ^1^H-NMR spectrum of Naringenin (Compound **1**); Figure S2: ^13^C-NMR spectrum of Naringenin (Compound **1**); Figure S3: COSY spectrum of Naringenin (Compound **1**); Figure S4: HMBC spectrum of Naringenin (Compound **1**); Figure S5: HMQC spectrum of Naringenin (Compound **1**); Figure S6: ESI-MS spectrum of Naringenin (Compound **1**) -ESI MS spectrum and +ESI MS spectrum, respectively; Figure S7: ^1^H-NMR spectrum of Apigenin (Compound **2**); Figure S8: ^13^C-NMR spectrum of Apigenin (Compound **2**); Figure S9: COSY spectrum of Apigenin (Compound **2**); Figure S10: HMBC spectrum of Apigenin (Compound **2**); Figure S11: HMQC spectrum of Apigenin (Compound **2**); Figure S12: ESI-MS spectrum of Apigenin (Compound **2**) -ESI MS spectrum and +ESI MS spectrum, respectively; Figure S13: ^1^H spectrum of Luteolin (Compound **3**); Figure S14: ^13^C spectrum of Luteolin (Compound **3**); Figure S15: COSY spectrum of Luteolin (Compound **3**); Figure S16: HMBC spectrum of Luteolin (Compound **3**); Figure S17: HMQC spectrum of Luteolin (Compound **3**); Figure S18: ESI-MS spectrum of Luteolin (Compound **3**) -ESI MS spectrum and +ESI MS spectrum, respectively.
