# Supplementary material for: From Ethnopharmacology to Active Compound: Effects of Traditional Plant Extracts on Varicose Vein-Related Enzymes and Isolation of Active Flavonoids from Helichrysum plicatum DC. subsp. plicatum
Source: Pharmaceuticals (Basel). 2025 Jun 19;18(6):926. doi: 10.3390/ph18060926 (PMC12195836; doi:10.3390/ph18060926)
Supplement: Supplementary file 1 [file pharmaceuticals-18-00926-s001.zip › pharmaceuticals-3715592-supplementary.pdf]

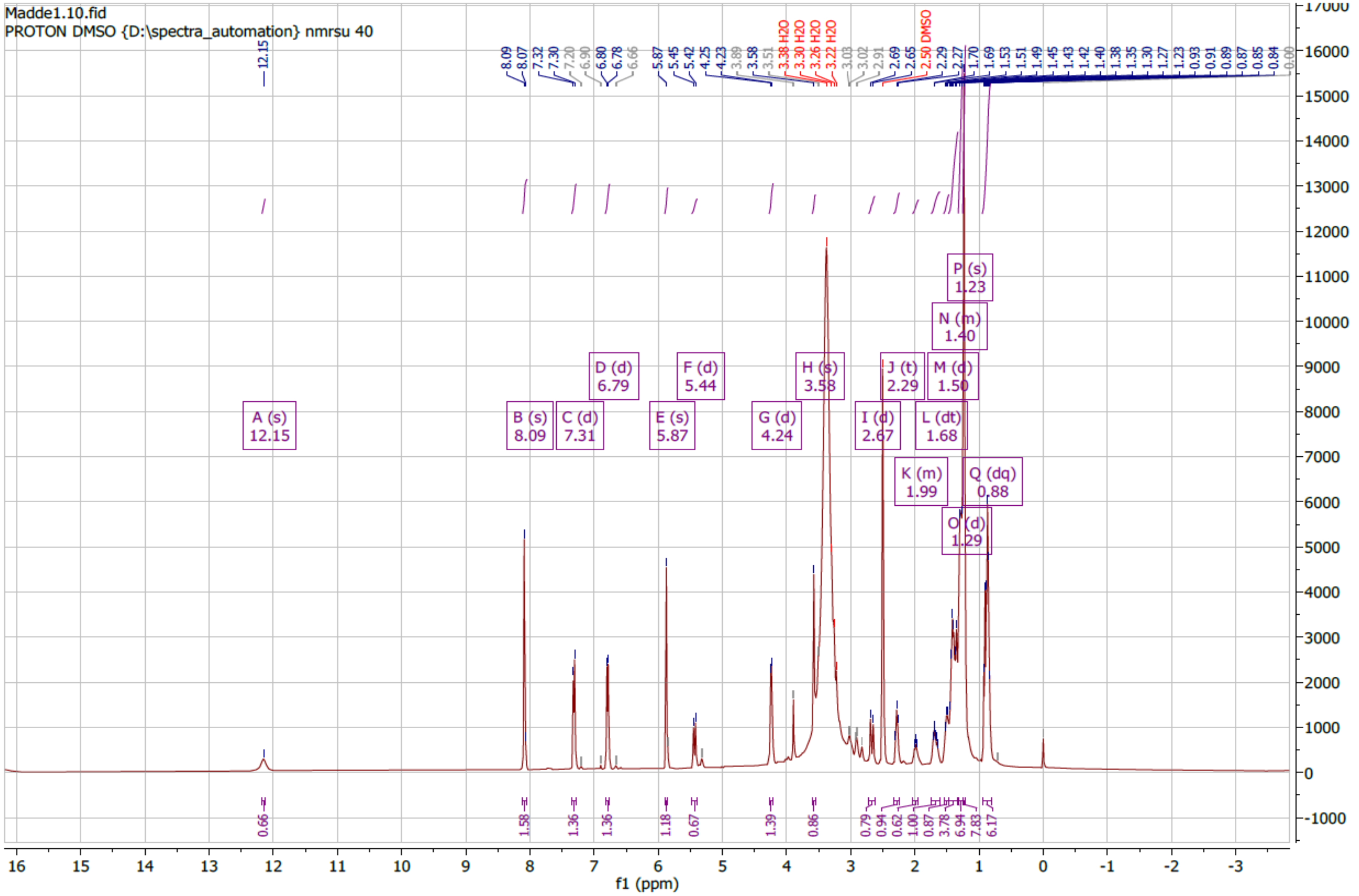

Figure S1. ^{1}H-NMR spectrum of Naringenin (Compound 1)

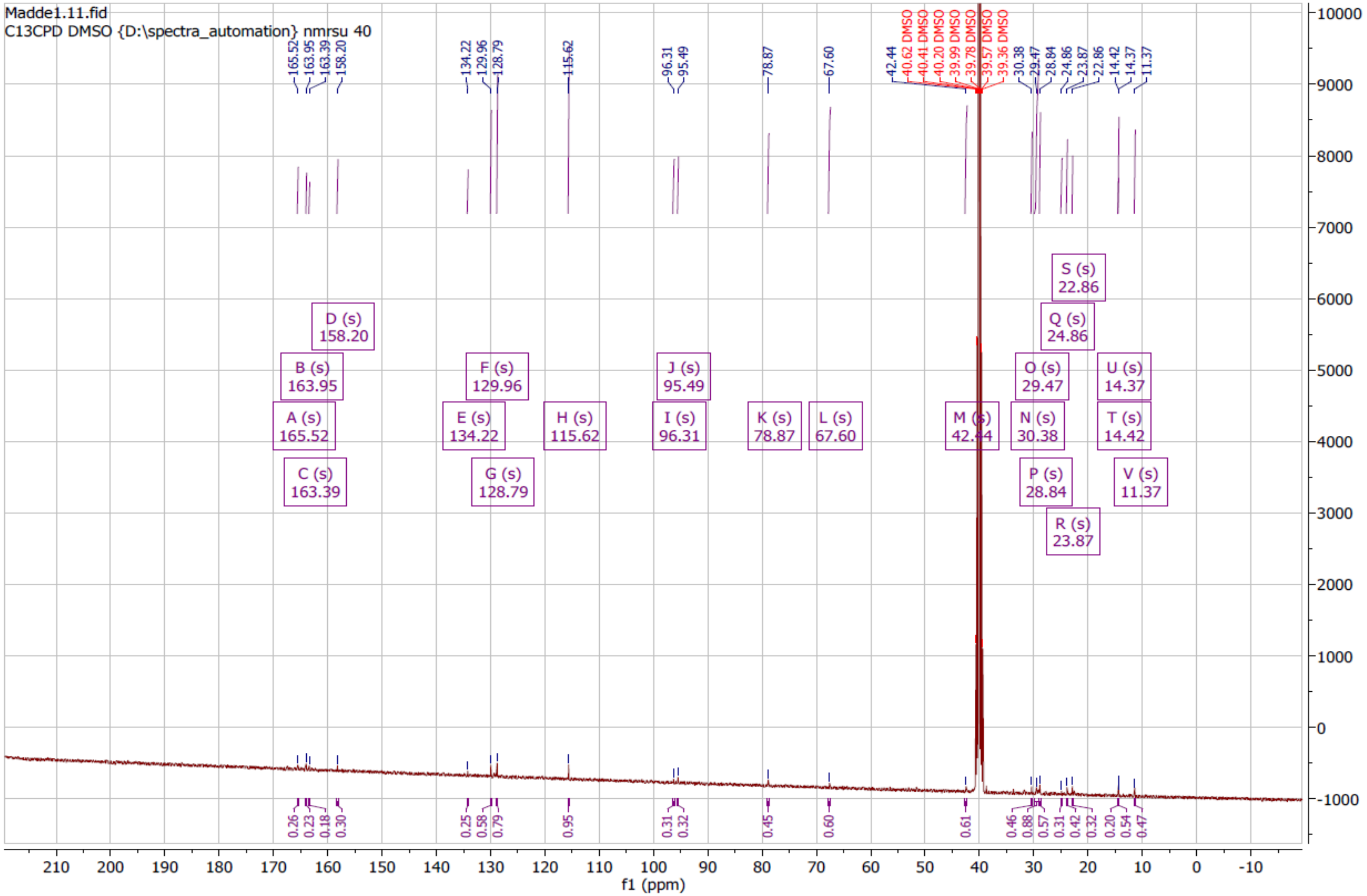

Figure S2. ^{13}C-NMR spectrum of Naringenin (Compound 1)

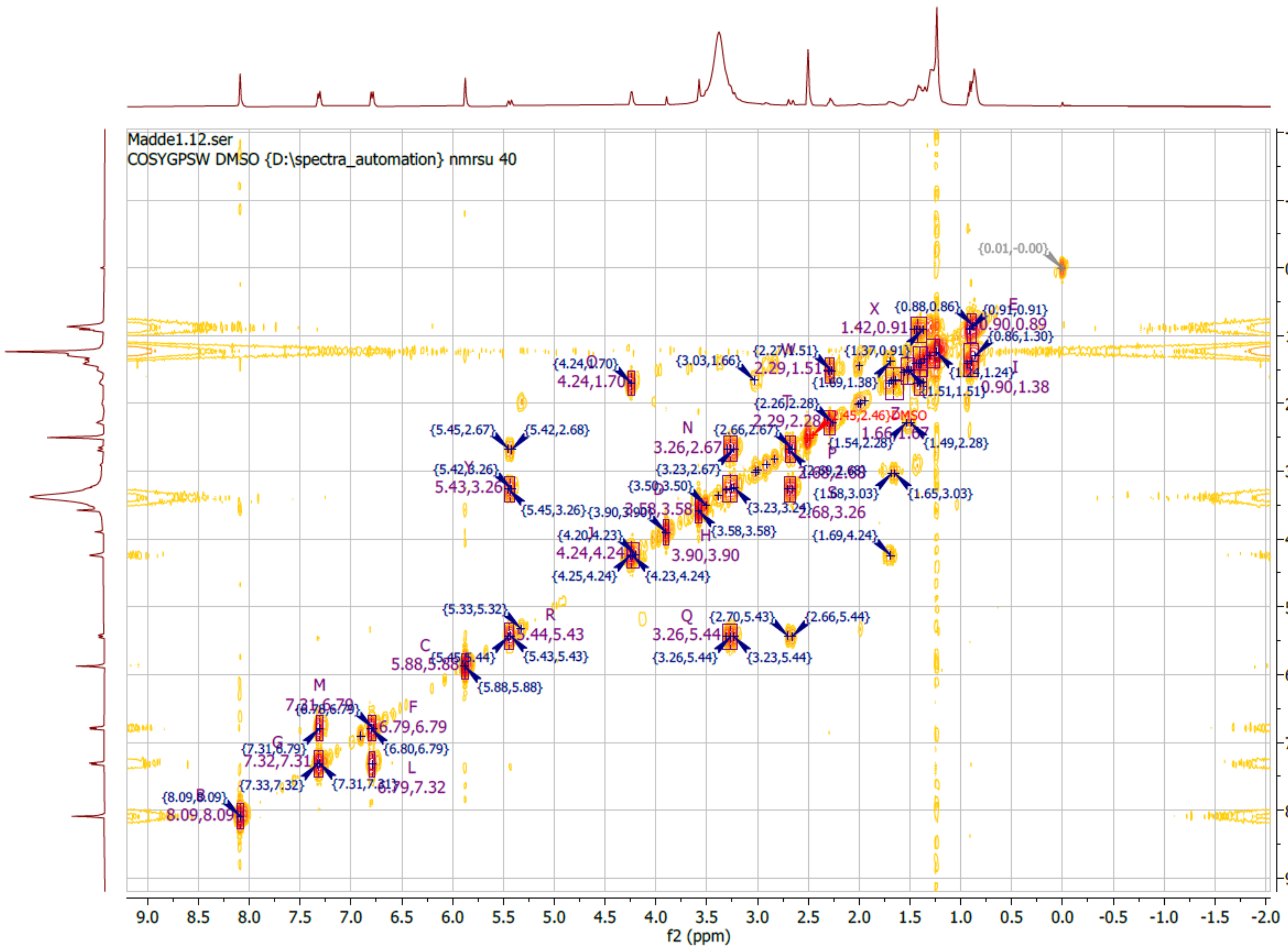

Figure S3. COSY spectrum of Naringenin (Compound 1)

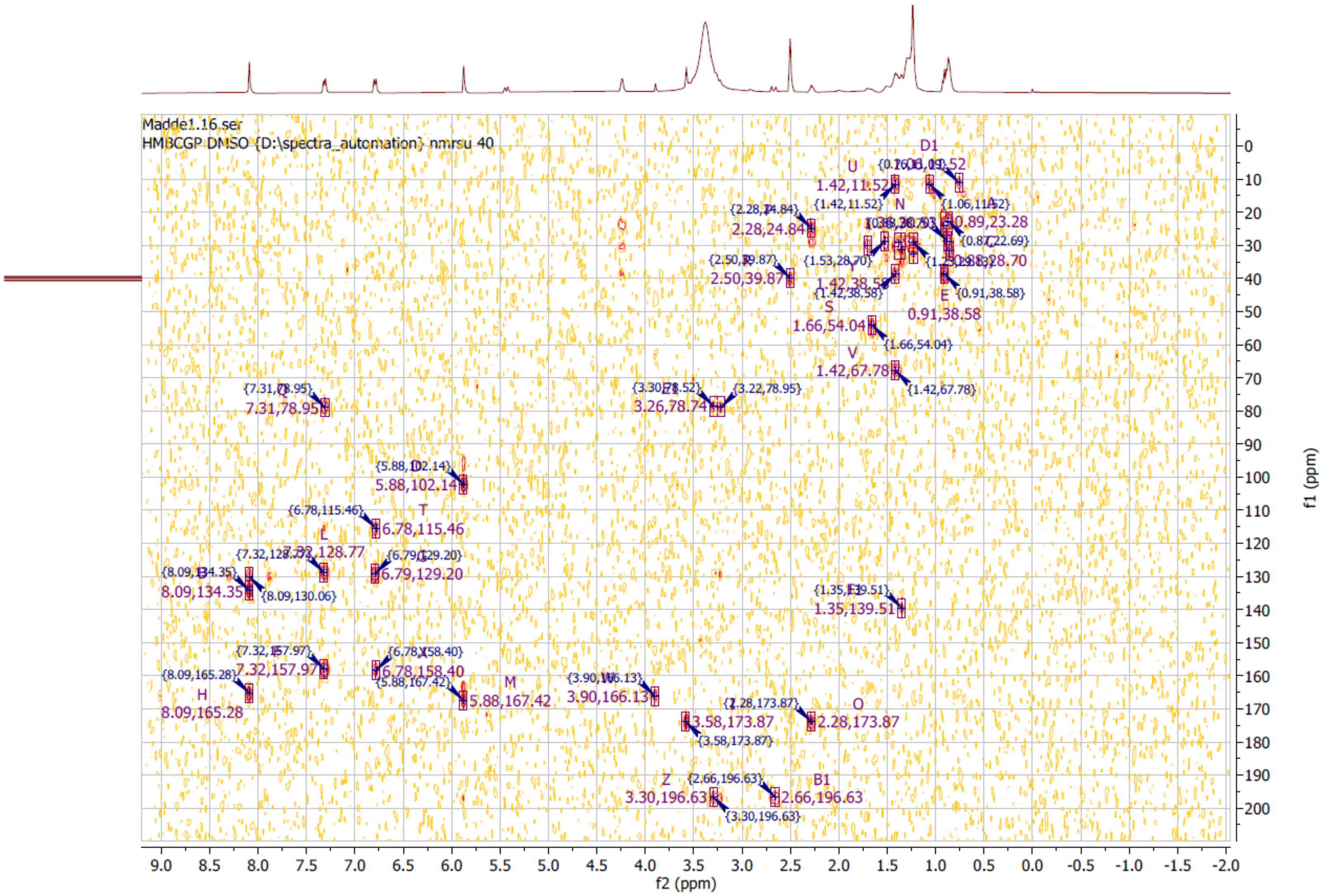

Figure S4. HMBC spectrum of Naringenin (Compound 1)

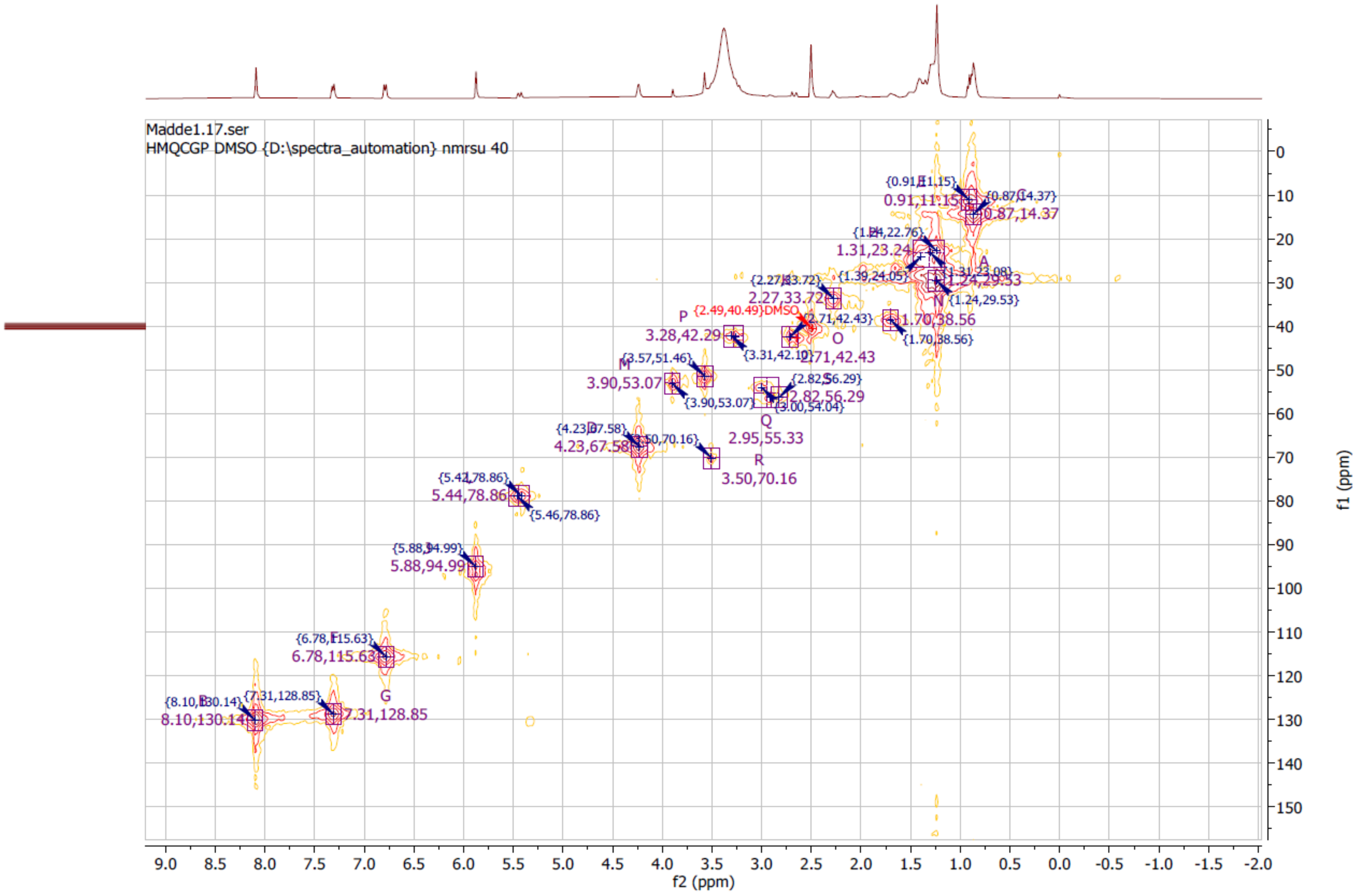

Figure S5. HMQC spectrum of Naringenin (Compound 1)

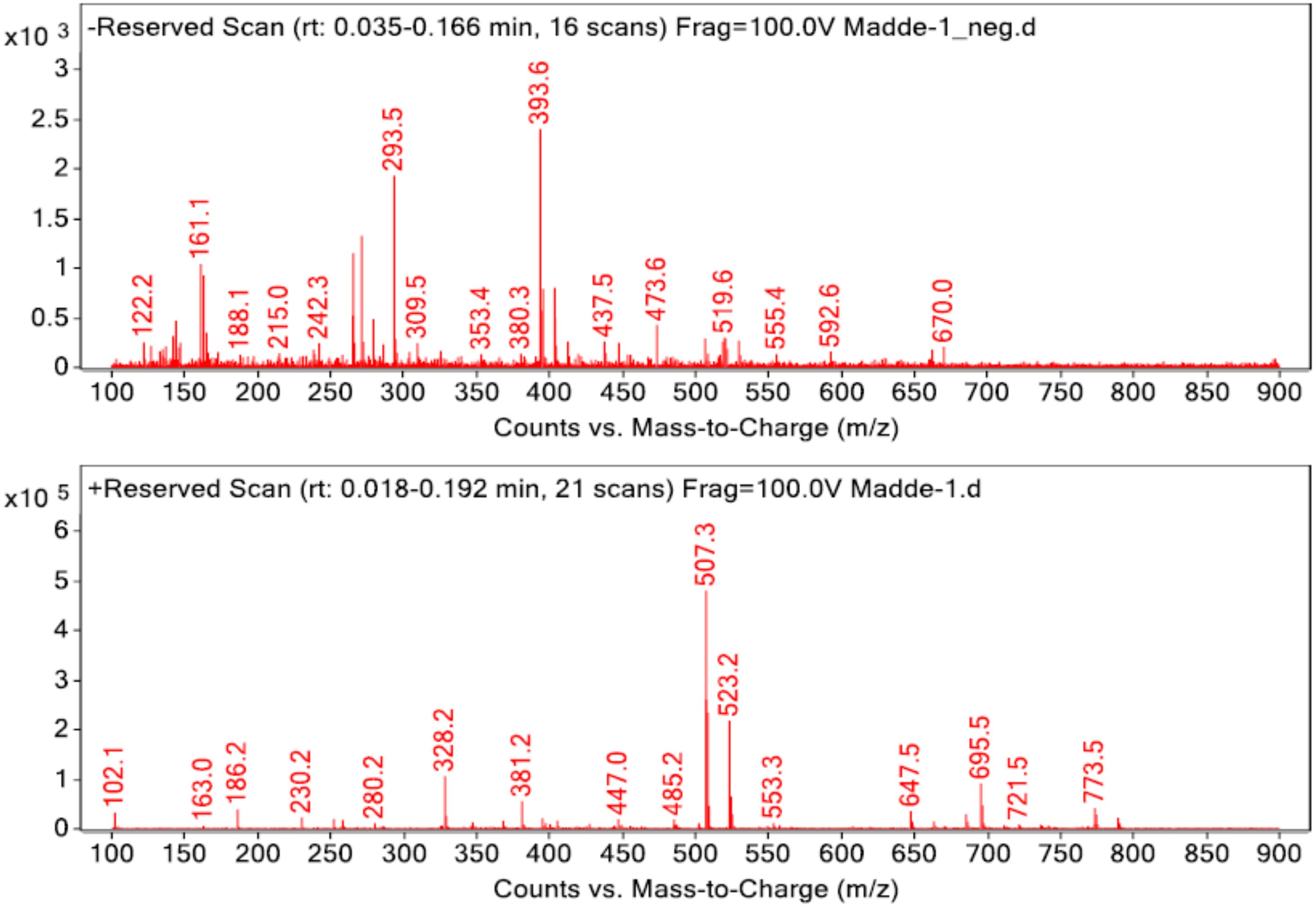

Figure S6. ESI-MS spectrum of Naringenin (Compound 1) -ESI MS spectrum and +ESI MS spectrum, respectively

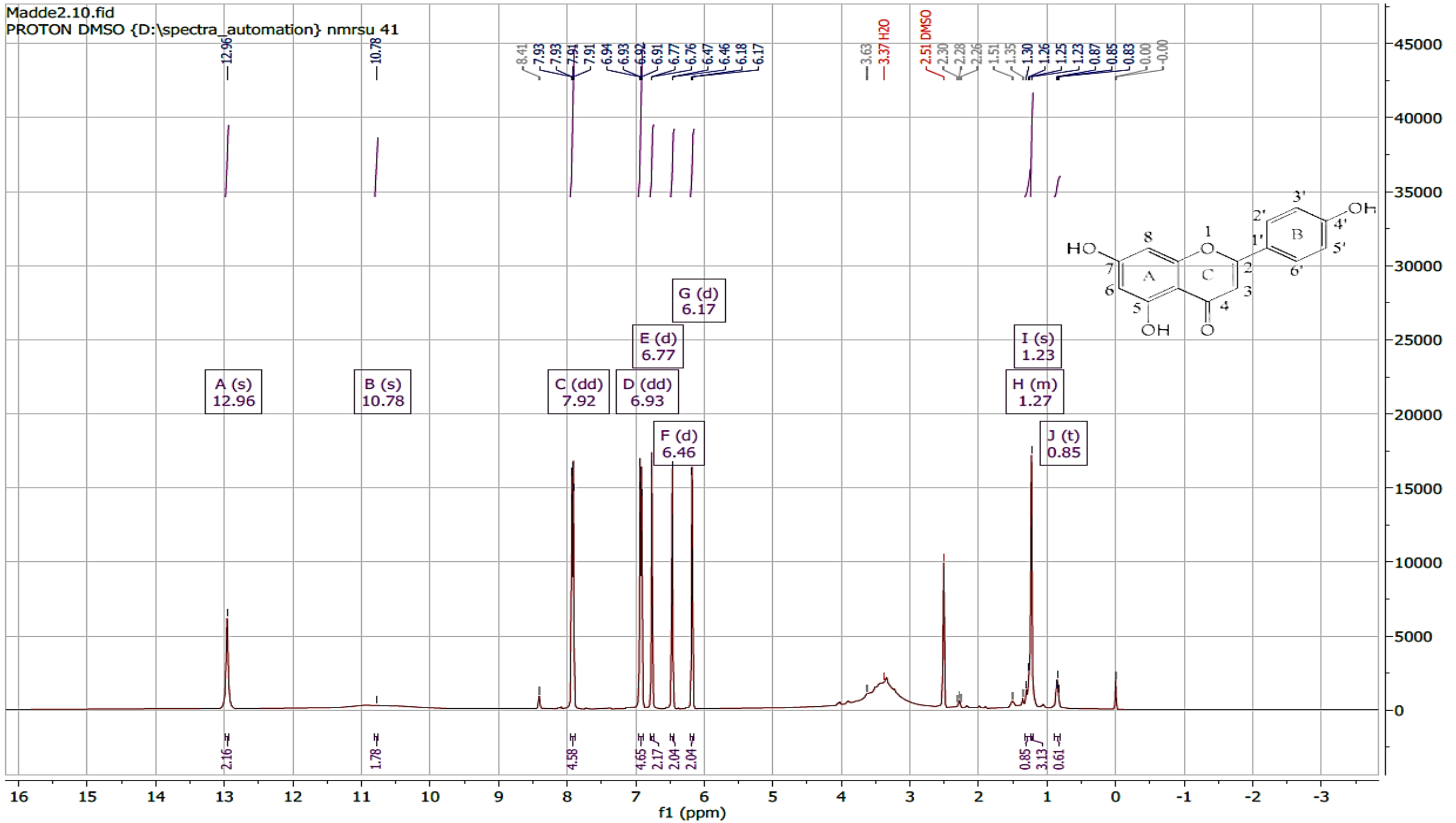

Figure S7. ^{1}H-NMR spectrum of Apigenin (Compound 2)

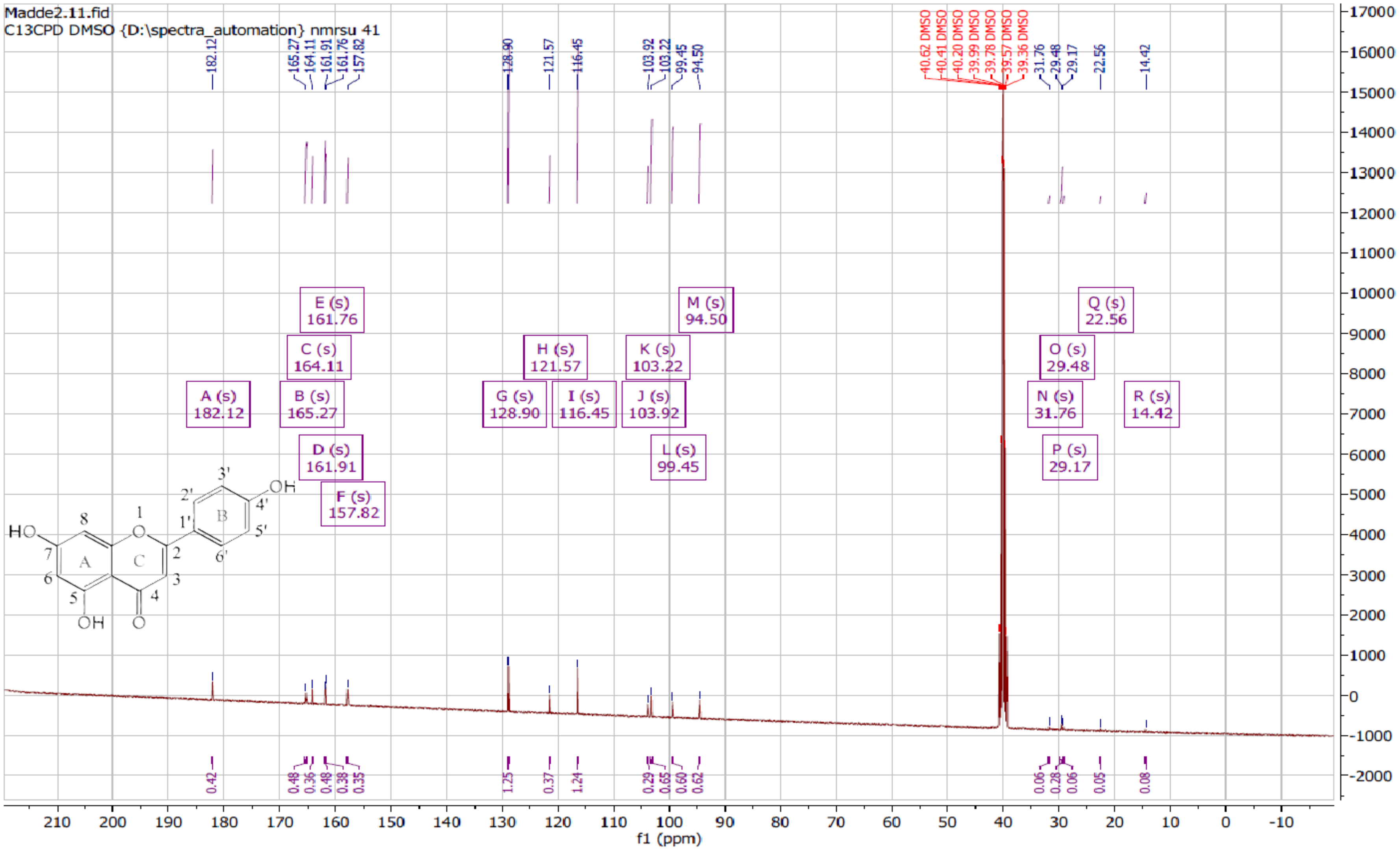

Figure S8. ^{13}C-NMR spectrum of Apigenin (Compound 2)

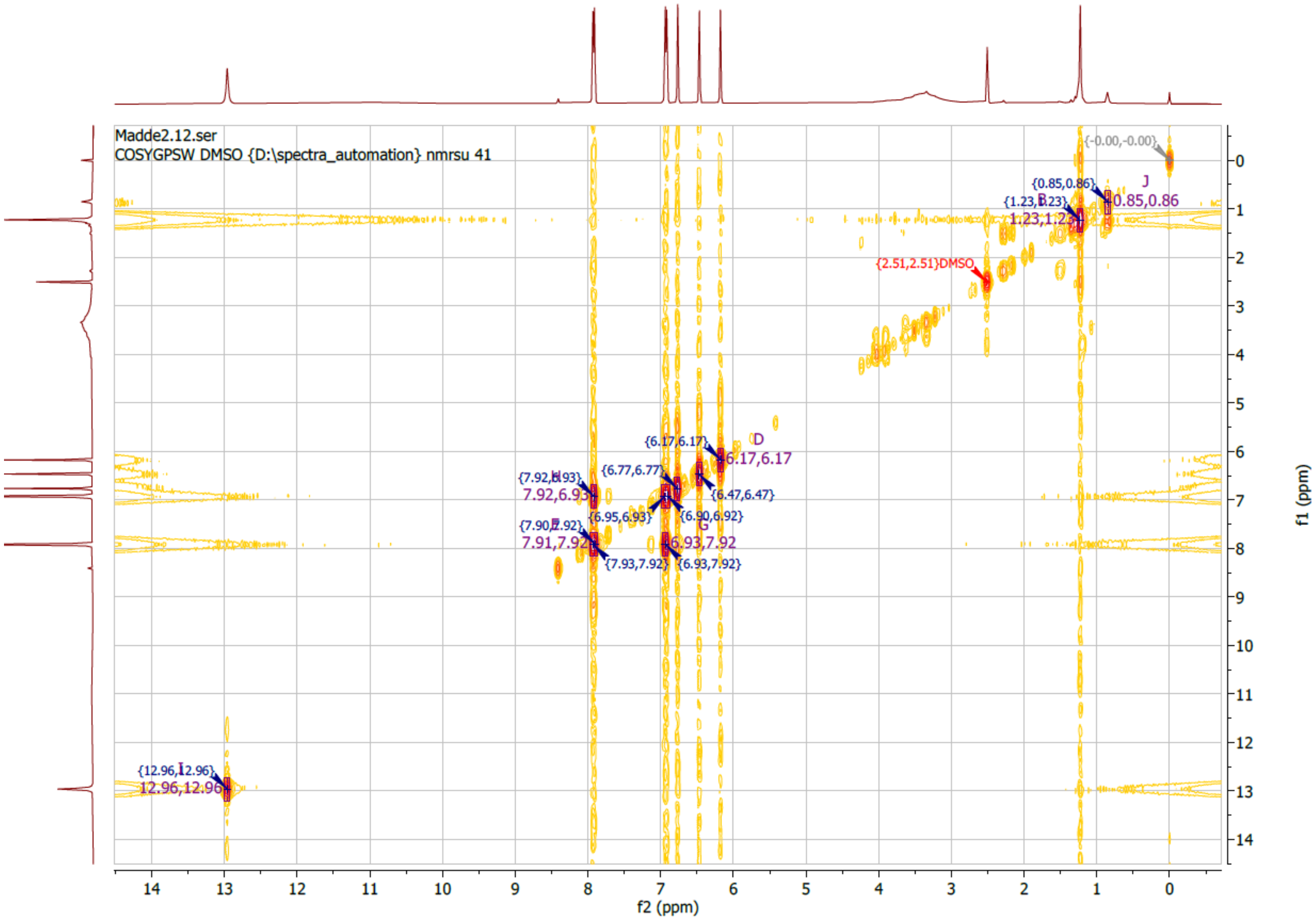

Figure S9. COSY spectrum of Apigenin (Compound 2)

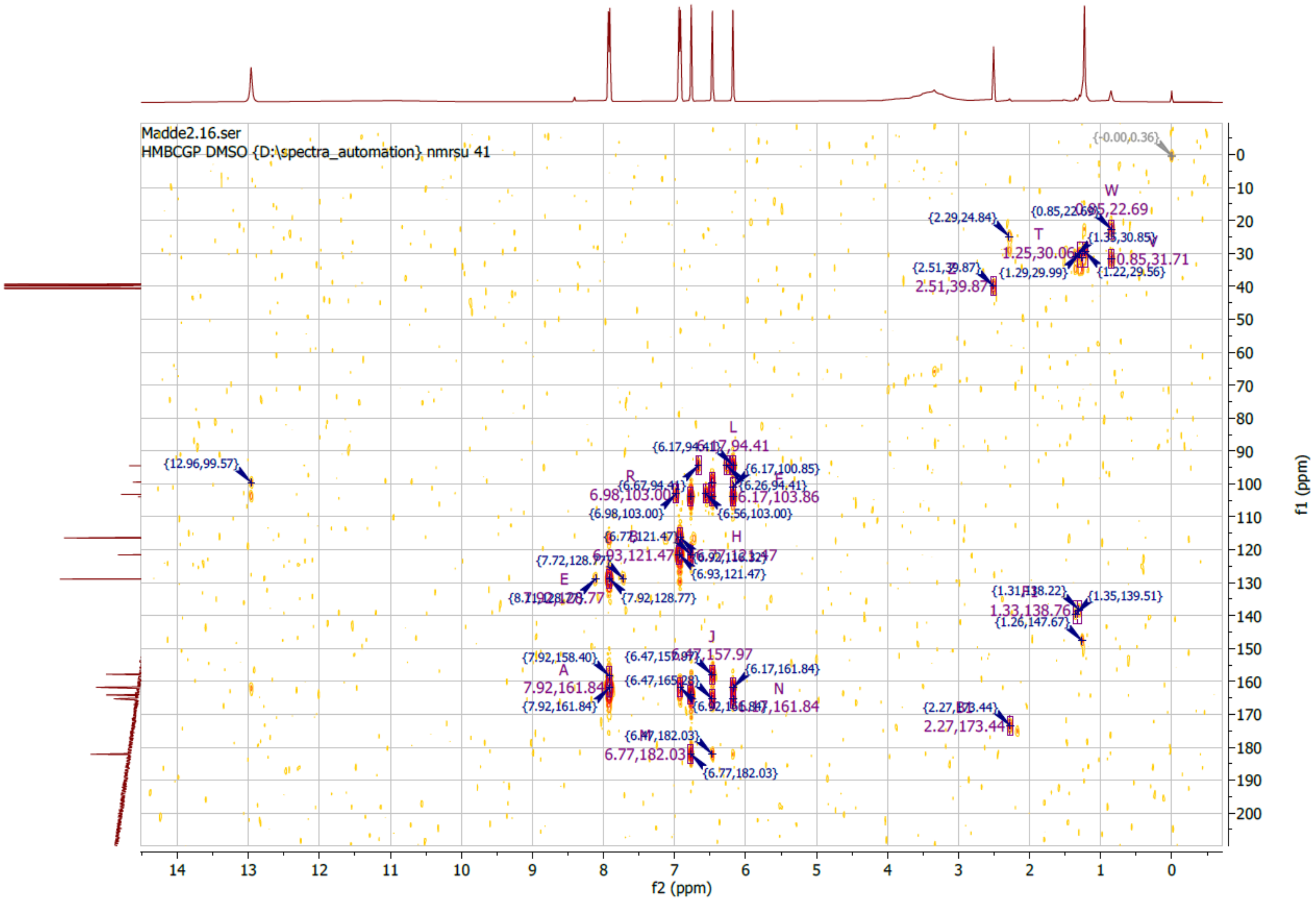

Figure S10. HMBC spectrum of Apigenin (Compound 2)

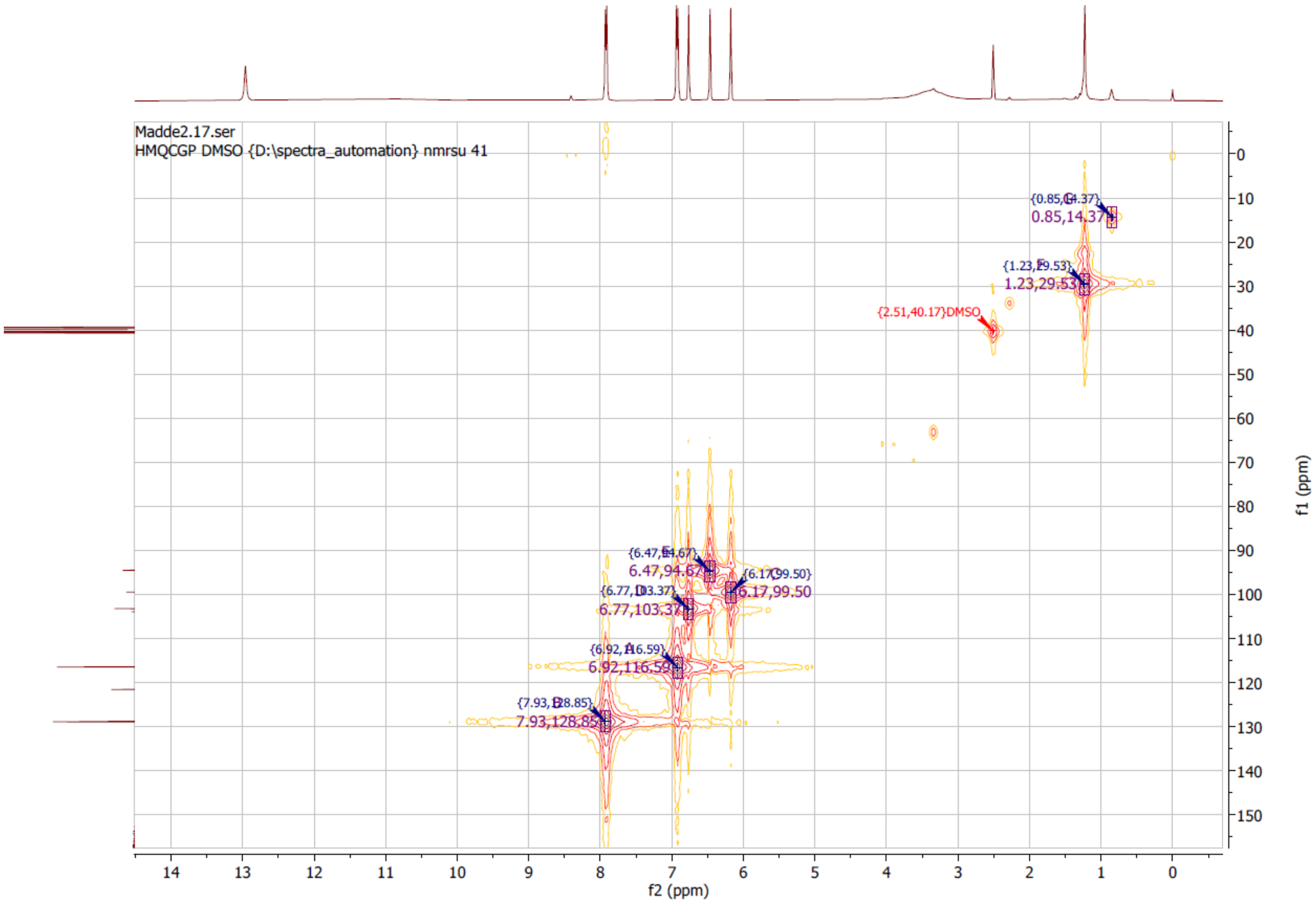

Figure S11. HMQC spectrum of Apigenin (Compound 2)

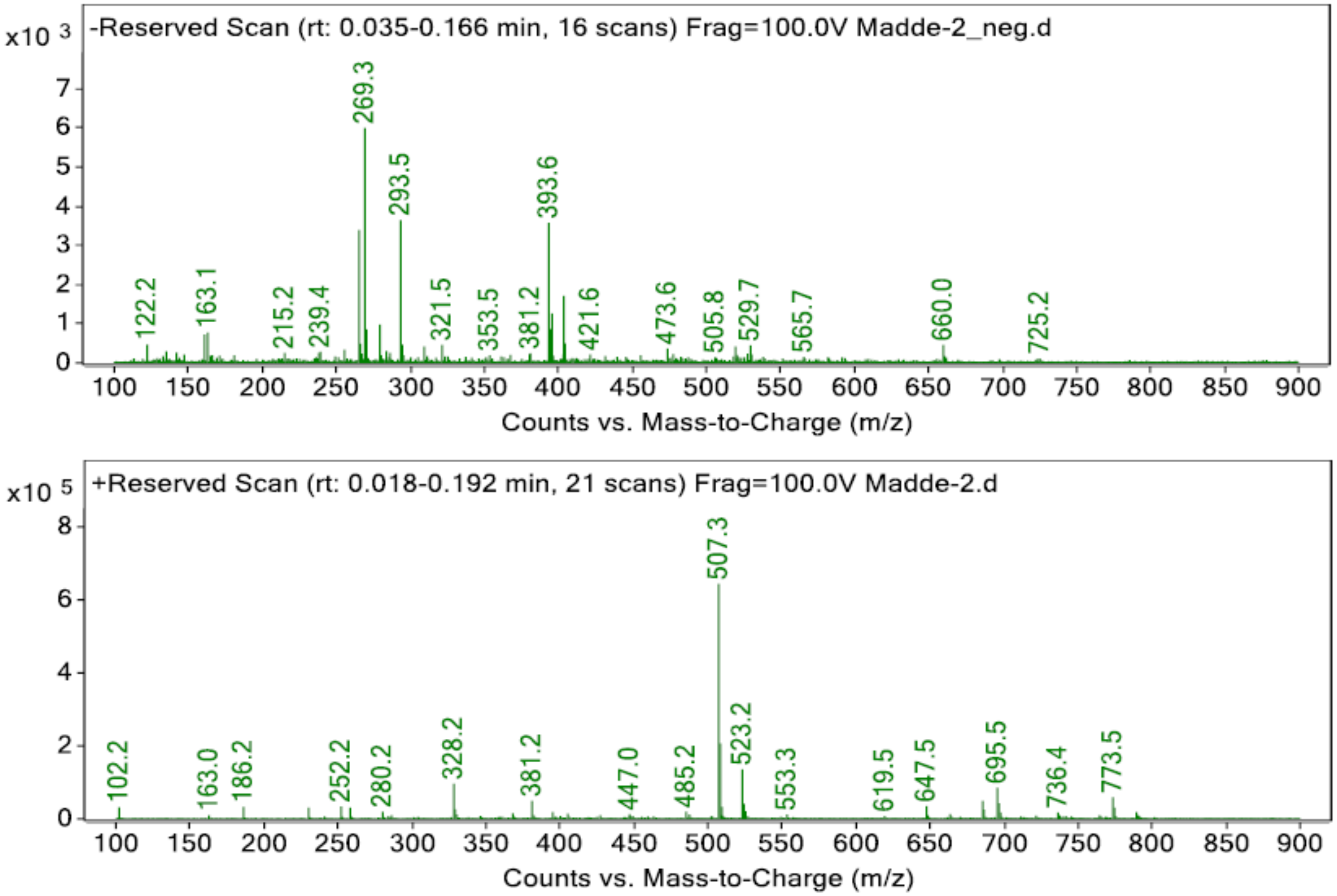

Figure S12. ESI-MS spectrum of Apigenin (Compound 2) -ESI MS spectrum and +ESI MS spectrum, respectively

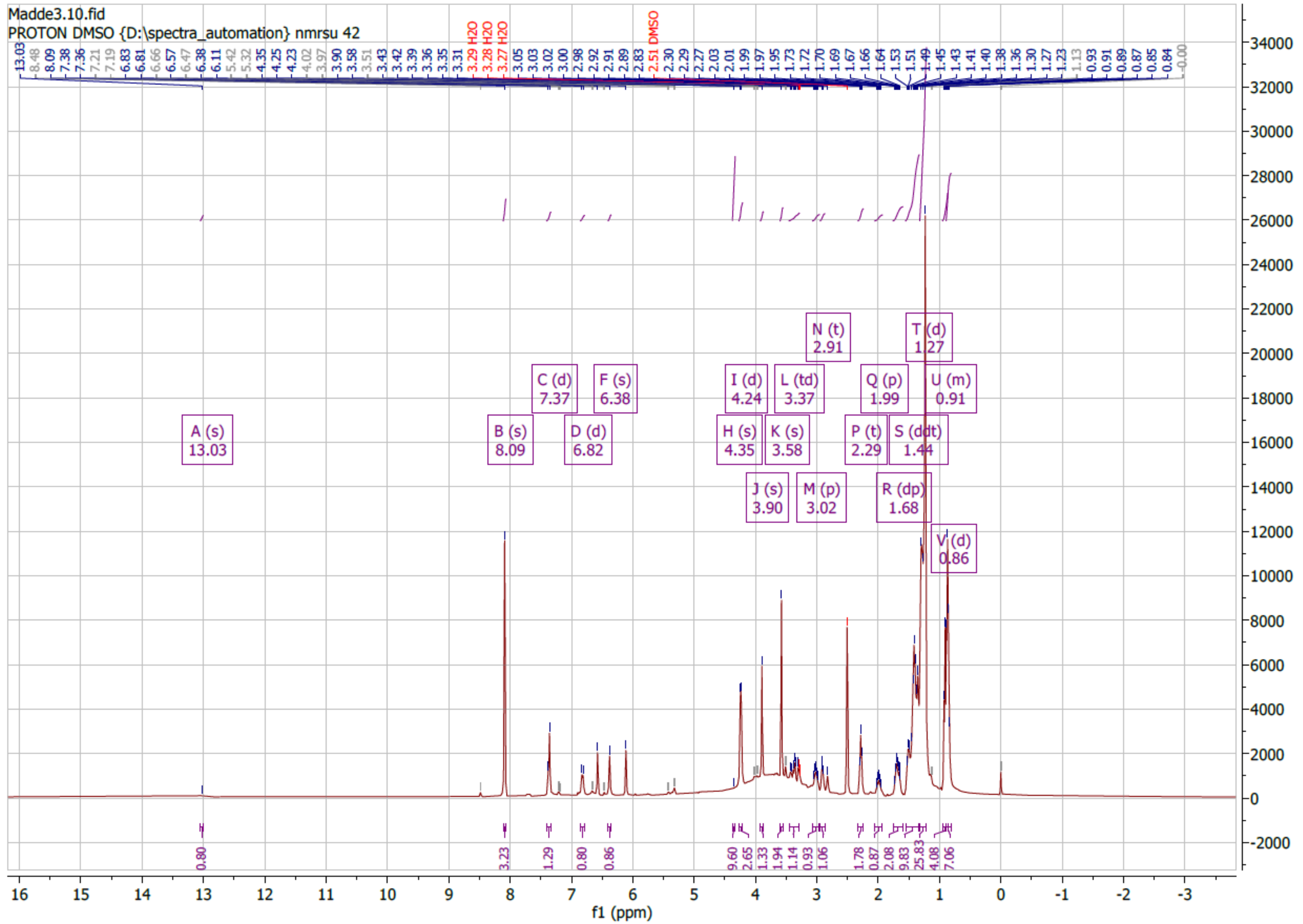

Figure S13. ^{1}H spectrum of Luteolin (Compound 3)

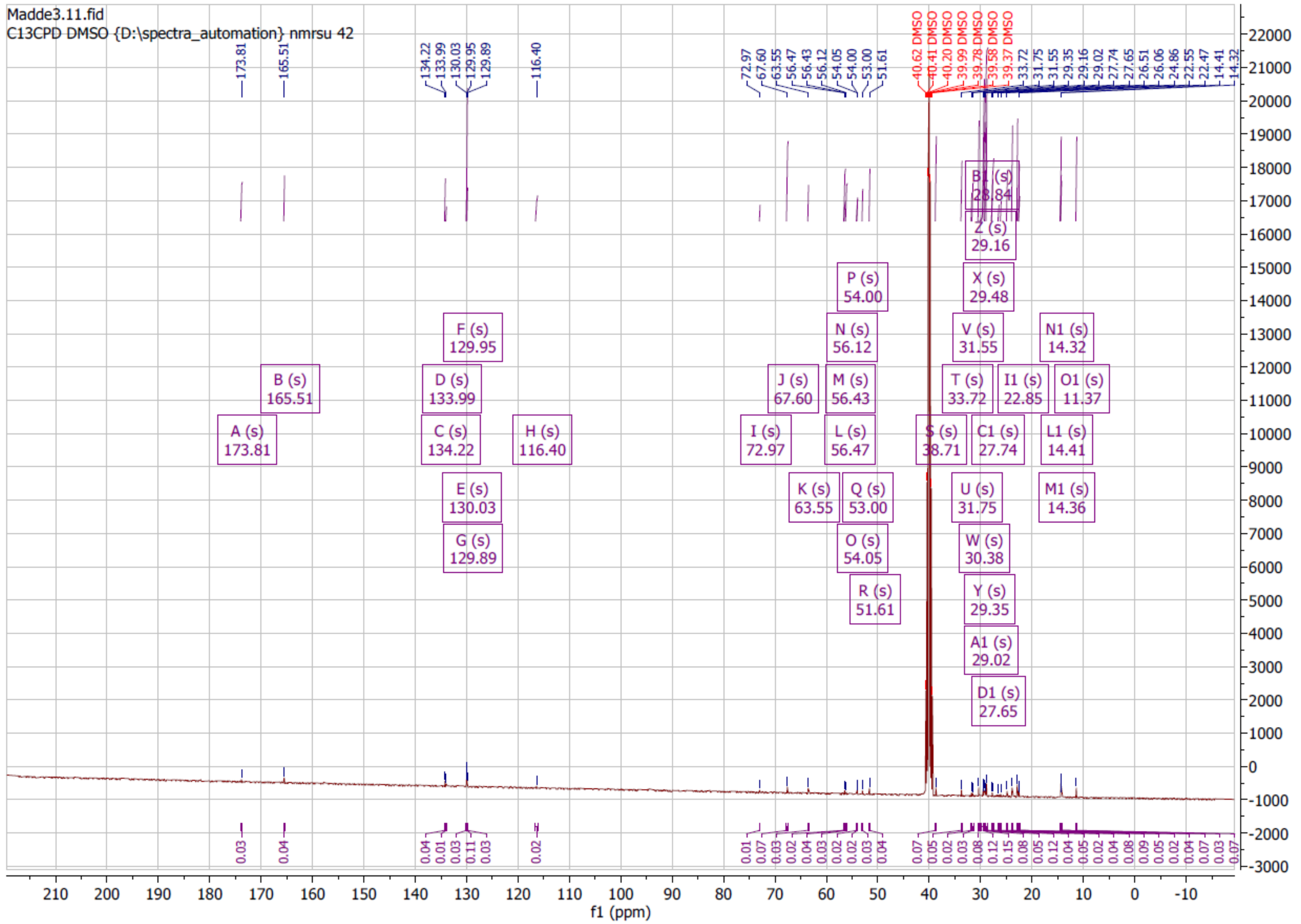

Figure S14. ^{13}C spectrum of Luteolin (Compound 3)

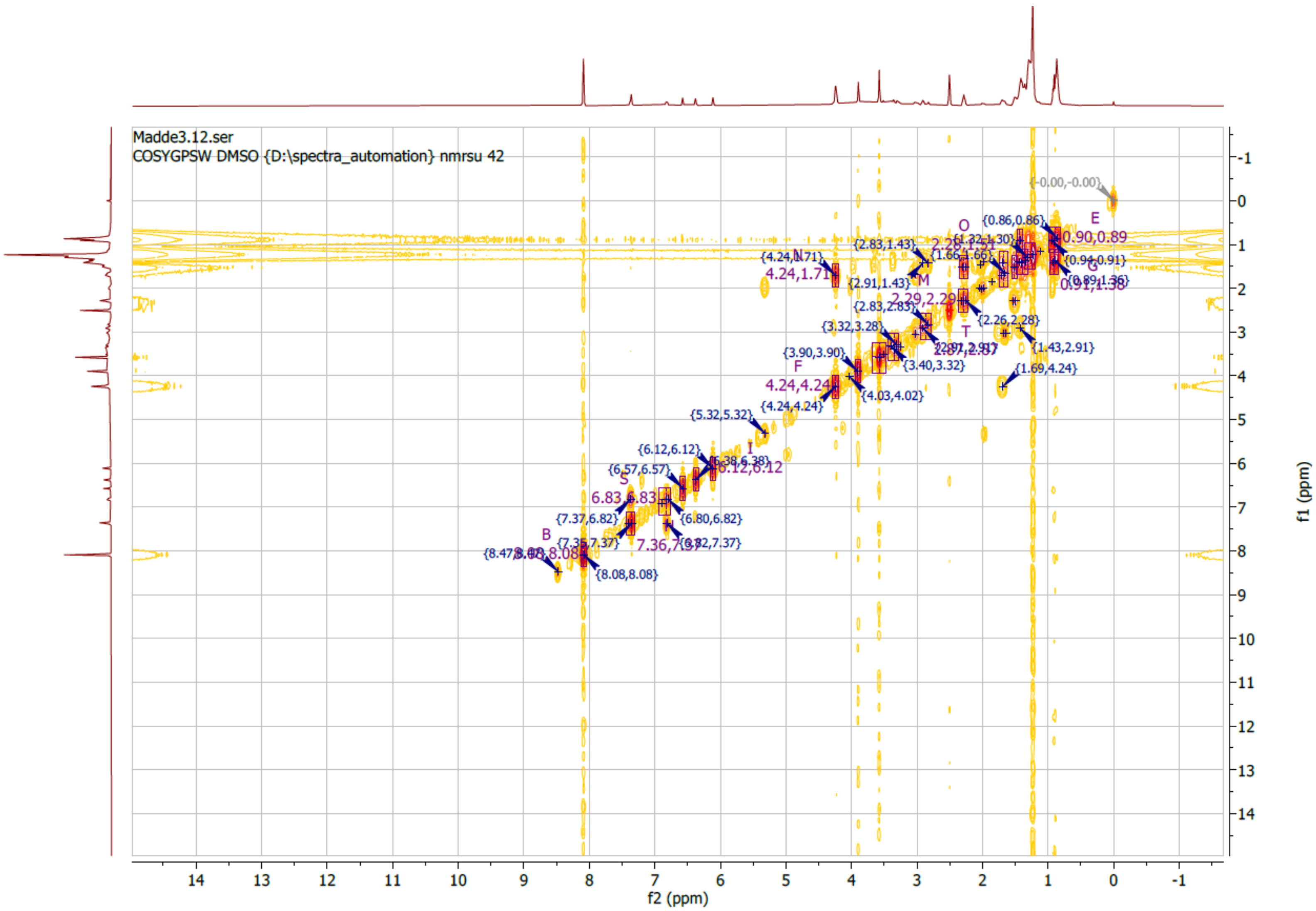

Figure S15. COSY spectrum of Luteolin (Compound 3)

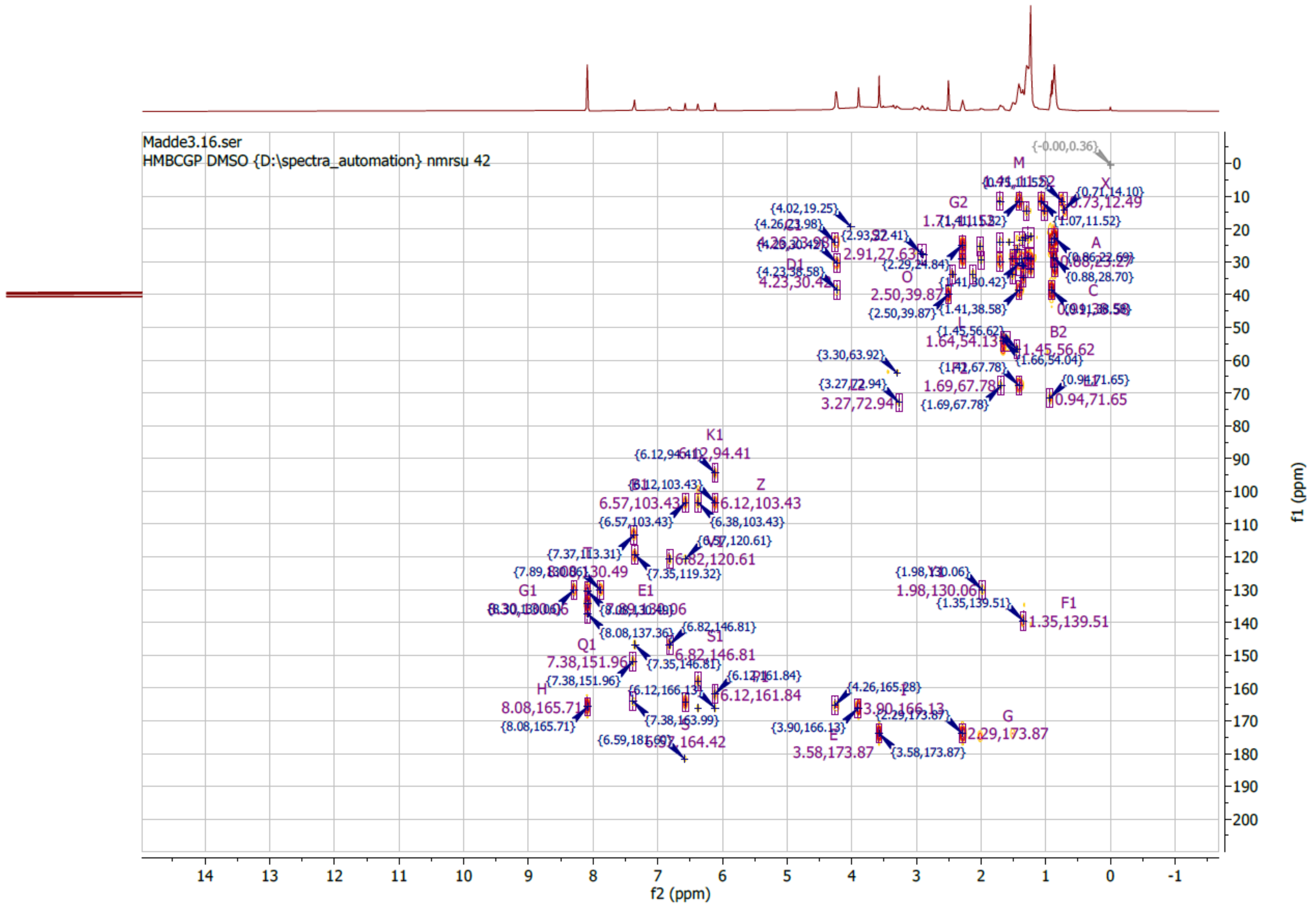

Figure S16. HMBC spectrum of Luteolin (Compound 3)

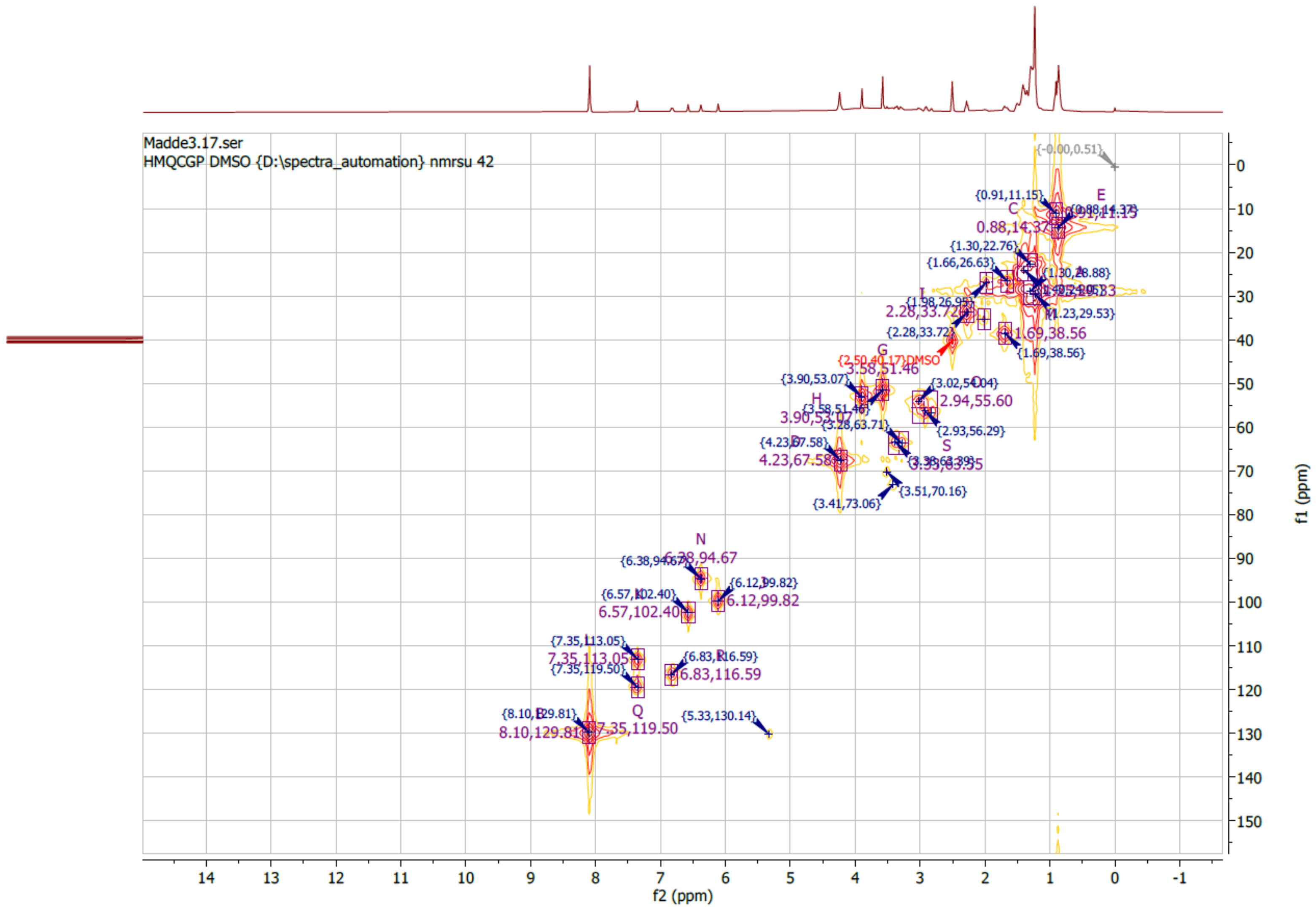

Figure S17. HMQC spectrum of Luteolin (Compound 3)

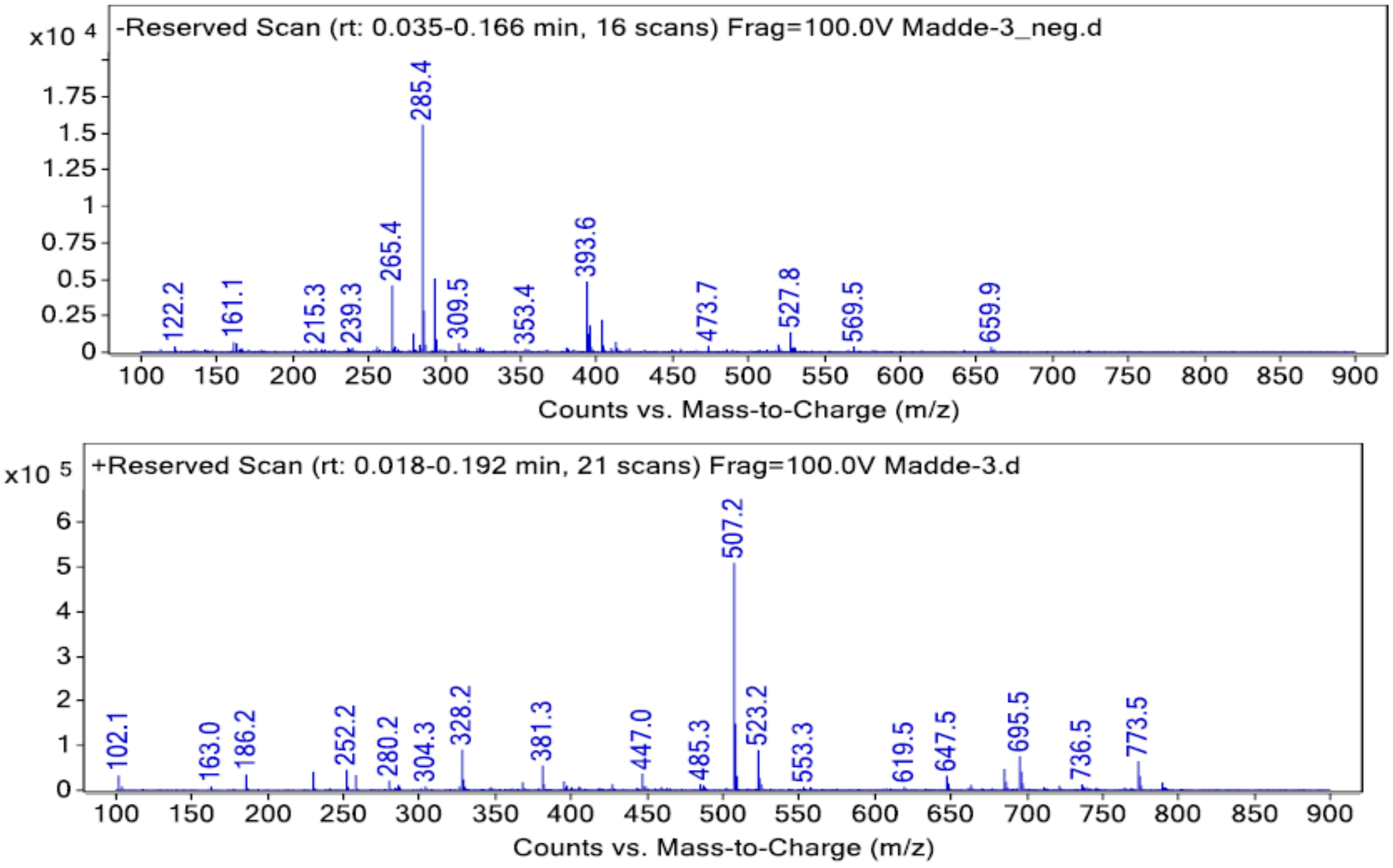

Figure S18. ESI-MS spectrum of Luteolin (Compound 3) -ESI MS spectrum and +ESI MS spectrum, respectively
